# Reorganization between preparatory and movement population responses in motor cortex

**DOI:** 10.1038/ncomms13239

**Published:** 2016-10-27

**Authors:** Gamaleldin F. Elsayed, Antonio H. Lara, Matthew T. Kaufman, Mark M. Churchland, John P. Cunningham

**Affiliations:** 1 Center for Theoretical Neuroscience, Columbia University, New York, New York 10032, USA; 2 Department of Neuroscience, Columbia University Medical Center, New York, New York 10032, USA; 3 Cold Spring Harbor Laboratory, Cold Spring Harbor, New York 11724, USA; 4 Grossman Center for the Statistics of Mind, Columbia University, 1255 Amsterdam Avenue, New York, New York 10027, USA; 5 David Mahoney Center for Brain and Behavior Research, Columbia University Medical Center, New York, New York 10032, USA; 6 Kavli Institute for Brain Science, Columbia University Medical Center, New York, New York 10032, USA; 7 Department of Statistics, Columbia University, 1255 Amsterdam Avenue, Room 1005 SSW, MC 4690, New York, New York 10027, USA

## Abstract

Neural populations can change the computation they perform on very short timescales. Although such flexibility is common, the underlying computational strategies at the population level remain unknown. To address this gap, we examined population responses in motor cortex during reach preparation and movement. We found that there exist exclusive and orthogonal population-level subspaces dedicated to preparatory and movement computations. This orthogonality yielded a reorganization in response correlations: the set of neurons with shared response properties changed completely between preparation and movement. Thus, the same neural population acts, at different times, as two separate circuits with very different properties. This finding is not predicted by existing motor cortical models, which predict overlapping preparation-related and movement-related subspaces. Despite orthogonality, responses in the preparatory subspace were lawfully related to subsequent responses in the movement subspace. These results reveal a population-level strategy for performing separate but linked computations.

## Abstract (web summary)

Single neuron responses are highly complex and dynamic yet they are able to flexibly represent behaviour through their collective activity. Here the authors demonstrate that population activity patterns of motor cortex neurons are orthogonal during successive task epochs that are linked through a simple linear function.

## Graphical abstract

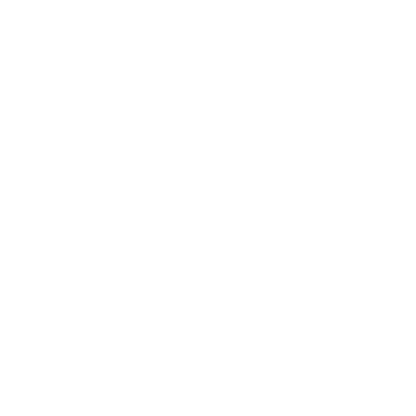

A major challenge faced by the brain is to perform different computations at different times using a fixed neural circuit. A canonical example occurs in the delayed-reach task, where a population of motor cortical neurons—spanning both primary and premotor cortex—participates in two processing stages. In the first stage, activity reflects neural processing during movement preparation^1^,^2^,^3^,^4^,^5^,^6^; we refer to this activity as the preparatory computation. In a subsequent stage, activity reflects neural processing underlying movement generation^7^,^8^,^9^,^10^; we refer to this activity as the movement computation. We have previously argued that one purpose of the preparatory computation is to produce a neural state (a collective pattern of activity across neurons) that determines how neural activity (and thus muscle activity) evolves during the movement computation^3^,^11^,^12^. There exist many further examples where the same neural population participates in multiple computations^13^,^14^,^15^,^16^,^17^,^18^,^19^,^20^,^21^,^22^, often with response properties that change in complex ways. How can a single population of neurons subserve one computation at one moment and another computation at the next moment? Does the change in computation have a population-level signature beyond the surface-level observation that neural responses have changed?

In the case of motor cortex, early models assumed a potentially straightforward relationship between preparatory and movement computations: preparatory activity might be a sub-threshold version of movement activity^23^,^24^. Subsequent studies indicated a more complex relationship^25^,^26^,^27^,^28^,^29^,^30^. In particular, single neurons typically exhibit different tuning (for example, a different relationship between firing rate and reach direction) during the preparatory and movement epochs^5^,^9^,^12^,^26^,^31^. Yet, despite this seemingly complex reorganization of responses, multiple lines of evidence argue that preparation and movement are mechanistically linked^3^,^6^,^32^,^33^,^34^,^35^,^36^,^37^.

Here we explore three basic population-level strategies that allow computations with reorganized neural responses, and which are compatible with the empirical complexity of single-neuron responses. The first arises when neural activity during two computations is independent at the population level. Sensory neurons commonly show independent tuning for key features (for example, direction and spatial frequency). By analogy, the motor cortex population might encode one set of variables during one computation and an independent set of variables during the next; an example in the motor system is maintaining posture and executing movement, where random changes in neural responses across these two behaviours strongly suggests the presence of independent computational processes^20^. This scenario yields a specific structure of activity across the population. During the first computation, neural activity occupies a particular subspace, defined by the response patterns and their correlations during that temporal epoch (Fig. 1a). Responses during the second computation are unrelated to those in the first computation and neural activity thus occupies an orthogonal subspace during the second epoch (Fig. 1b). The fact that responses are independent during the two computations is also reflected in the pairwise correlation structure, which changes completely between epochs (heatmaps in Fig. 1a,b).

A second type of population-level structure arises when the two successive computations are overlapping. In this scenario, both computations share a neural subspace (Fig. 1a,c). Neural responses may be simple or complex, but in either case the underlying space—and thus the correlation structure—is, to a large degree, preserved across computations. A simple example comes from the oculomotor system where pre-saccadic neural activity is a sub-threshold form of saccade-related activity^38^,^39^. A more complex example is the two-interval discrimination model of Machens *et al*.^40^ in which sensory information is loaded, sustained and subsequently used to render a discrimination, all within a single neural subspace. In a more nuanced example that is germane to the present work, Kaufman *et al*.^41^ found that preparatory activity avoids key output-potent dimensions within the activity space occupied during movement. This finding was interpreted as indicating that preparatory activity occupies a subspace of the larger space occupied during the movement computation. The models of Churchland *et al*.^11^ and Sussillo *et al*.^42^ employ this same strategy: responses during the preparatory and movement computations share some neural dimensions, but preparatory activity avoids causing premature movement by avoiding a few key dimensions that directly influence muscle activity. This series of studies thus assumes overlapping computations.

A third type of population-level structure arises when two successive computations are orthogonal but linked: different computations take place in orthogonal subspaces (as in the independent case), yet are still linked and related (as in the overlapping case). In this scenario (Fig. 1a,d) the neural subspace would change completely, yet there would still be a consistent relationship between the neural states across the two computations (for example, the ordering of conditions is the same in Fig. 1a,d). The orthogonal but linked strategy has been suggested in the context of rodent parietal cortex during a multi-sensory decision^21^, based on the observation that the neural dimensions most strongly occupied during movement (a whole-body movement towards a reward port) were quite modestly occupied during the preceding multi-sensory decision.

Whether motor cortex displays independent, overlapping or orthogonal-but-linked structure cannot be inferred from existing results. Complex changes in single-neuron tuning^12^,^20^,^31^ are potentially consistent with all three scenarios, as is the finding that preparatory activity avoids output potent dimensions linked to muscle activity^41^. Yet, as systems neuroscience increasingly examines the responses of large neural populations, it becomes critical to characterize the structure of those responses^43^,^44^. Different circuit-level mechanisms will produce different population structures and it can be critical to know which structure is present when analysing data. For example, a common analysis approach is to train a decoder to predict key experimental variables during one epoch or context and use that decoder to interpret data from another epoch or context^14^,^45^,^46^,^47^. This approach is appropriate in the case of overlapping structure, but not for independent or orthogonal-but-linked structures. Finally, the interpretation of neural correlations depends on the population structure. It is often assumed that groups of correlated neurons cooperate to perform a similar function, but is that cooperation a static property or is it specific to a particular computation?

In the case of motor cortex, the majority of analyses and models have assumed that preparatory and movement computations are overlapping. This assumption is made by essentially all models of motor cortex activity, including dynamical systems models^11^, network models^48^ and rise-to-threshold models^23^,^24^. As discussed above, even for models that use a null space for preparation^11^,^41^, the neural dimensions occupied during preparation are contained within, and thus overlapping with, the movement dimensions. The assumption of overlapping subspaces is also implicitly made when the same population readout—for example, a population vector^49^,^50^ or a population firing rate^4^—is applied to neural responses across an entire trial. Yet, some studies have suggested independent preparatory and movement computations^27^. This could occur if preparatory activity reflects one set of variables (for example, target location) while movement-epoch activity reflects a different set of variables (for example, muscle activity or proprioceptive feedback). Although potentially compatible with existing data, the possibility of orthogonal-but-linked computations has not previously been considered in the context of preparation and movement in motor cortex.

Here we report that the motor cortex employs the orthogonal-but-linked strategy. Motor cortex effectively acts as two different circuits, with largely unrelated correlation structure during preparation and movement. Yet, a simple linear transformation accounts for the flow of activity from the preparatory subspace to the movement subspace. Thus, despite single-neuron response complexity, the population-level structure is surprisingly simple: motor cortex exploits separate subspaces when performing successive computations, yet those computations can be linked by a flow of activity from one subspace to the other, just before movement onset. These findings indicate a degree of flexibility that was not displayed or predicted by existing motor cortical models. Our results thus reveal a population-level strategy that is present when an area flexibly changes computations, but is not yet accounted for by existing theories.

## Results

### Motor cortex responses during delayed-reach task

We trained two rhesus macaque monkeys on a centre-out delayed-reach task similar to the paradigm used in prior studies^5^,^9^,^51^ (Fig. 2a). Our task had similar conditions and geometry to our previous studies^11^,^52^: reaches were made between a central touch point and a radial target displayed on a monitor placed in front of the monkey, whereas the hand was tracked optically. Each trial began when the monkey touched a central touch point. After a brief hold period (450–550 ms), a target appeared in one of eight radially arranged locations (Fig. 2a, dashed circles). After a variable delay (0–1,000 ms), a go-cue instructed a reach towards the target. Only trials with a delay >450 ms were analysed. Both monkeys successfully waited during the delay period and executed brisk, accurate reaches (Fig. 2b) with short reaction times (monkey B: 252±46 s.d. ms, monkey A: 237±39 s.d. ms).

**Figure 1:**
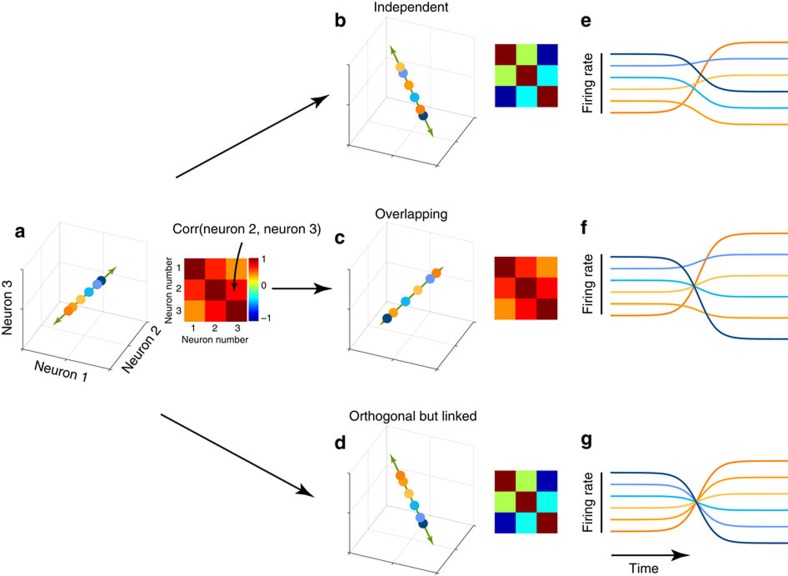
Illustration of neural states and across-condition correlations for three hypothetical neurons during two successive computations. (**a**) Activity of three hypothetical neurons involved in the first of two successive computations. Each axis represents the firing rate of one neuron and each dot represents the neural state for one of six conditions. The activity of the three neurons occupies a subspace (green line) of the full space of possible states. The heatmap shows the cross-condition correlation matrix: large values indicate that the relevant pair of neurons has activity that covaries across conditions. (**b**) Population structure for the independent strategy. Responses occupy a different neural subspace from that in **a** and the correlation structure is changed completely. Further, there is no particular relationship between the ordering of conditions in **b** versus that in **a**. (**c**) Population structure for the overlapping strategy. The across-condition pattern of neural activity changes across the computations: the set of dots is now in a different order. However, the subspace occupied by neural activity remains the same and the correlation structure is thus preserved. (**d**) Population structure for the orthogonal-but-linked strategy. Neural activity occupies a different subspace and thus the correlation structure changes. Yet, unlike the situation in **b**, the ordering of conditions is lawfully related to that in **a**. In this example the ordering is identical, but activity is in a new subspace. (**e**–**g**) Firing rates as a function of time for neuron 2 for the three types of population structure described above. The colour of each trace indicates the condition identity. In time, the first half of each trace corresponds to the first computation and the second half corresponds to the second computation. Regardless of the type of population structure, the response of this neuron changes in complex ways from one computation to the other.

**Figure 2:**
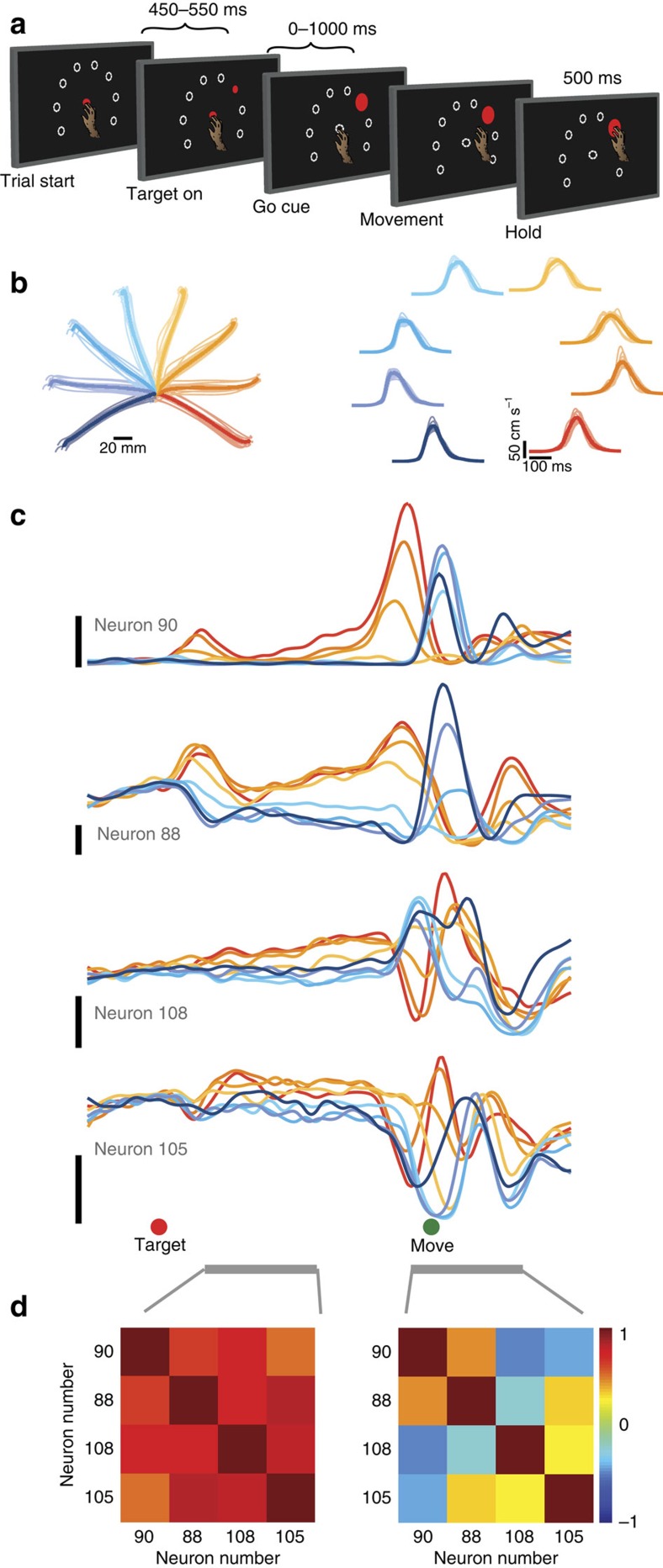
Task and example neurons. (**a**) Events in the delayed-reach task. Monkeys made reaches to one of eight possible targets displayed on a monitor. Dashed circles (not visible to the monkey) indicate the possible reach-target locations. (**b**) Reach trajectories and velocity profiles for monkey B. Thick traces denote the average trajectory across all recording sessions, thin traces denote the average trajectories for 15 randomly chosen sessions. (**c**) Responses of four example neurons. Each trace is the trial-averaged firing rate during a reach in one of the eight directions. Trace colour indicates reach direction (shown in **b**). Red dot indicates target onset time. Green dot indicates movement onset time. Grey horizontal bars denote the 300ms preparation and movement epochs. Black vertical bars denote 20 spikes per sec. (**d**) Correlation matrices for the four example neurons during preparatory (left) and movement (right) epochs.

We recorded single-neuron responses (127 neurons from monkey B, 98 from monkey A) from the primary motor cortex (M1) and the immediately adjacent region of dorsal premotor cortex (PMd). We computed firing rates by filtering spike trains using a Gaussian kernel and averaging across trials. We defined the preparatory epoch as a 300 ms interval beginning 150 ms after target onset. This epoch started after preparatory activity had developed and ended before neural activity transitioned from movement preparation to execution. We defined the movement epoch as a 300 ms interval beginning 50 ms before movement onset, which is approximately when muscle activity begins to change. Most neurons responded during both epochs and exhibited temporally complex responses (Fig. 2c). The four example neurons in Fig. 2c illustrate that the condition with the highest firing rate during the preparatory epoch was rarely the condition with the highest firing rate during the movement epoch. Indeed, most neurons showed a wide range of relationships between tuning during the preparatory and movement epochs. A key question arises: if two neurons have similar response patterns in the preparatory epoch, do they also have similar response patterns during the movement epoch? If response patterns change in similar ways, it would suggest that subspaces overlap across epochs (Fig. 1a,c). If response patterns change in different ways, it may suggest orthogonal subspaces (Fig. 1b,d). It is noteworthy that whether subspaces are overlapping or orthogonal does not hinge on whether individual neurons display complex response patterns (Fig. 1e–g), but on whether sets of neurons that share a response pattern in one epoch tend to also share some response pattern in the other epoch. To examine this question, the four example neurons in Fig. 2c were selected to have strongly correlated preparatory-epoch response patterns: firing rates are highest for rightwards reaches and lowest for leftwards reaches. The first two example neurons continued to exhibit correlated response patterns during the movement epoch. This maintained similarity is consistent with the general idea that neurons that are correlated are functionally connected and perform a similar role, which will generalize from one epoch to another. However, this pattern was exceptional: most other neuron pairs did not maintain their similarity across epochs. For example, neuron 90 and neuron 108 had very different patterns of movement-epoch activity. As a result, the correlation matrix, which quantifies the correlation between all pairs of the four neurons, largely changed between preparatory and movement epochs (Fig. 2d). We next examined whether this was true at the level of the whole population.

### Correlation structure during preparation and movement

**Figure 3:**
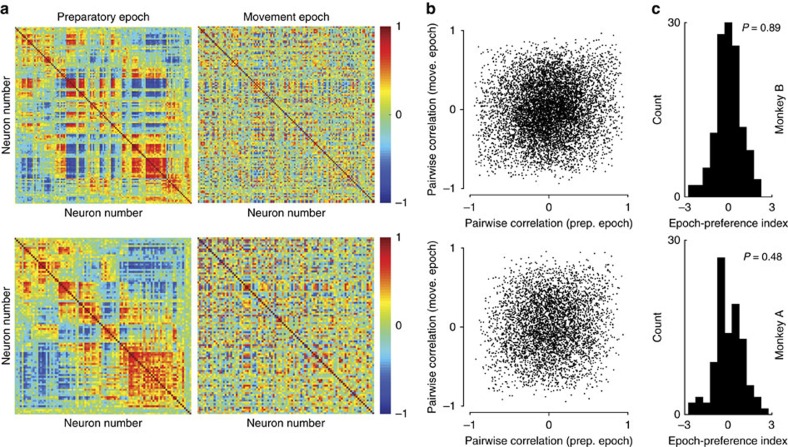
Preparatory-epoch and movement-epoch correlation structure for all neurons. (**a**) Preparatory-epoch (left) and movement-epoch (right) correlation matrices for all neurons for monkey B (top) and monkey A (bottom). Each entry in the matrix gives the degree to which the response pattern was similar for the two neurons during that epoch. The order of neurons is the same for the preparatory-epoch matrix and the movement-epoch matrix. (**b**) The correlation for each neuron pair during the movement epoch plotted against the correlation for the same pair during the preparatory epoch. (**c**) Histogram of epoch-preference index, which quantifies the strength of neural activity during the preparatory epoch compared with the strength of neural activity during the movement epoch (see text). Positive values indicate that a neuron is more selective during the preparatory epoch and negative values indicate that a neuron is more selective during the movement epoch. The distributions are not significantly bimodal (Hartigan's dip test; dip statistic monkey B=0.024; *P*=0.89, and dip statistic monkey A=0.036; *P*=0.48).

We calculated the correlation matrices for the full neural population from each monkey (Fig. 3a). To aid visualization, we chose a neuron ordering to highlight the structure in the preparatory-epoch correlation matrix and used the same ordering for the movement-epoch correlation matrix. For both monkeys, the correlation structure changed markedly between preparatory and movement epochs. Indeed, when we plotted the entries of the movement-epoch correlation matrix against the entries of the preparatory-epoch correlation matrix, we found little relationship (Fig. 3b; *R*^*2*^=0.11±0.02 for monkey B, *R*^*2*^=0.09±0.03 for monkey A). In other words, for a given pair of neurons, the similarity of responses during the preparatory epoch is largely uninformative regarding the similarity of responses during the movement epoch. This is particularly striking as the two epochs are separated by only a few hundreds of milliseconds.

One potential explanation for the change in correlation structure is that separate sets of neurons are active during the preparatory and movement epochs. To investigate this possibility, for each neuron, we calculated an epoch-preference index (Methods). A neuron that is active only during the preparatory (movement) epoch would have a positive (negative) index. A neuron that is active during both epochs would have an index of 0. The presence of separate preparatory-only and movement-only neurons would result in a bimodal distribution of indices. The distributions peaked near zero (Fig. 3c) with no evidence for bimodality (Hartigan's dip test; *P*=0.89 for monkey B, *P*=0.48 for monkey A). Indeed, neurons with strong activity during only one epoch were rare. Thus, preparatory and movement computations are not performed by separate populations of neurons. We also repeated the analysis from Fig. 3a,b including only neurons with strong reach-direction tuning during both epochs. The change in correlation structure was undiminished (Supplementary Fig. 1).

### Different computations occur in separate subspaces

The results above are inconsistent with the hypothesis that the population response occupies an identical subspace during preparation and movement; in that case, the correlation matrix would have remained similar across epochs as in Fig. 1a,c (although the matrix need not have been identical, it would have been similar in the technical sense of sharing a similar eigen-basis; see below). Given the lack of similarity, there are three alternative possibilities. First, the dimensions occupied during the preparatory epoch could overlap with, but be a subset of, the dimensions occupied during movement. This proper subspace strategy is employed by the model of Churchland *et al*.^11^ and was the interpretation of Kaufman *et al*.^41^ who found that preparatory activity occupies dimensions that are orthogonal to dimensions in which neural activity resembles muscle activity (Supplementary Fig. 2a). In that interpretation, movement is implicitly gated by preparatory activity avoiding a few key movement-epoch dimensions: the output-potent dimensions. Yet, the preparatory dimensions were still assumed to overlap with (that is, be contained within) the movement dimensions. A second, related possibility is that the two subspaces could be partially overlapping, with some preparatory-epoch-only dimensions, some movement-epoch-only dimensions and some overlapping dimensions (Supplementary Fig. 2b). The third possibility is that the preparatory-epoch and movement-epoch subspaces could be fully orthogonal (Supplementary Fig. 2c). No previous work has investigated these possibilities.

**Figure 4:**
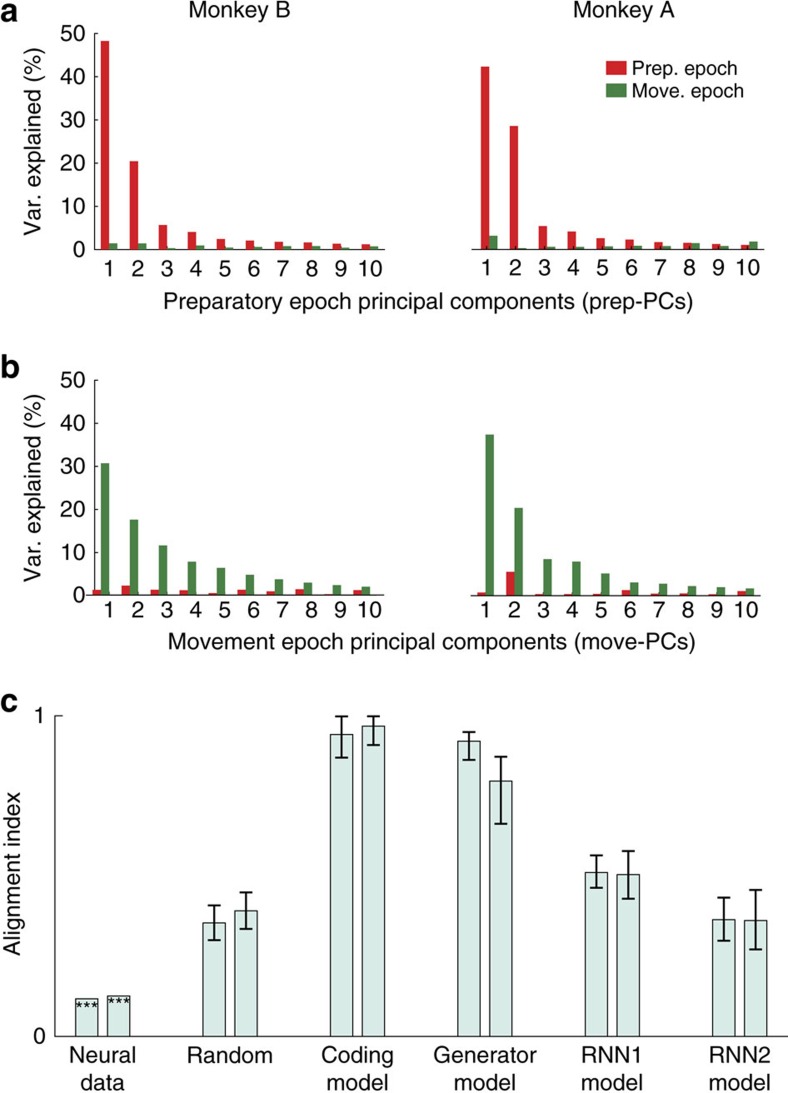
Percentage of variance explained by preparatory and movement principal components (prep-PCs and move PCs). (**a**) Percentage of preparatory-epoch data variance (red bars) and movement-epoch data variance (green bars) explained by the top ten prep-PCs. (**b**) Percentage of preparatory-epoch data variance and movement-epoch data variance explained by the top ten move-PCs. (**c**) Alignment index for neural, random and model data. For each pair, the two bars correspond to data from monkey B and A, or to simulated data sets based on real data from two monkeys. Bars labelled ‘random' correspond to the distribution of indices expected from random dimensions within the space occupied by the data. The last four pairs of bars were obtained from simulated data generated from a coding model, a pattern generator model, a non-normal RNN (RNN1) and a regularized RNN (RNN2). Stars for the neural data bars denote a significantly lower index relative to both random and to all models (*P*<0.001, one-tailed test). For random data and models, the bars show the median index across multiple bootstrap resamples and error bars denote the 95% confidence interval (based on the distribution obtained via bootstrap).

The above possibilities can be distinguished using principal component analysis (PCA): the principal components are the eigenvectors of the unnormalized correlation matrix (that is, the covariance matrix). If preparatory-epoch and movement-epoch dimensions are orthogonal, the preparatory-epoch principal components (prep-PCs) will capture little movement-epoch data variance and the movement-epoch principal components (move-PCs) will capture little preparatory-epoch data variance. To identify the prep-PCs and move-PCs, we performed PCA separately on the preparatory and movement-epoch responses. By definition, the top ten prep-PCs captured a large amount of preparatory-epoch data variance (Fig. 4a) and the top ten move-PCs captured a large amount of movement-epoch data variance (Fig. 4b). However, the top ten prep-PCs captured very little movement-epoch data variance and the top ten move-PCs captured very little preparatory-epoch data variance. This finding reveals that motor cortex does not use the preparatory-epoch dimensions during the movement epoch and, conversely, movement-epoch dimensions are not used during the preparatory epoch.

The above finding indicates that the prep-PCs and move-PCs are close to orthogonal. We quantified the degree of orthogonality by calculating an alignment index: the preparatory-epoch data variance captured by the top ten move-PCs, normalized by the preparatory-epoch data variance captured by the top ten prep-PCs (Methods). If the prep-PCs and move-PCs do not overlap, the index will be 0; if they overlap completely, the alignment index will be 1. For both monkeys, the index was close to zero (Fig. 4c), indicating that preparatory-epoch and movement-epoch responses explore two near-orthogonal sets of dimensions.

Are the prep-PCs and move-PCs more strongly misaligned than expected if both were random sets of dimensions within the space occupied by neural activity^53^? The higher the dimensionality of that space, the easier it is to find two random sets of near-orthogonal dimensions by chance. To assess the baseline misalignment that results from partitioning the neural space into sets of random dimensions, we randomly sampled sets of ten preparatory-epoch dimensions and ten movement-epoch dimensions based on the full covariance structure of the data (Methods and Supplementary Fig. 3). The resulting alignment index was higher than the index from neural data (Fig. 4c). Thus, in the neural data, preparatory-epoch dimensions and movement-epoch dimensions are more strongly misaligned than expected if they were random draws from the space occupied by the data. This is particularly surprising, because one might have expected that the two sets of preparatory and movement dimensions would be actively aligned (more overlapping than expected by chance) because of the presumed relation between preparation and movement computations. In fact, they appear to be actively misaligned (closer to orthogonal than expected by chance). Do current models of motor cortex responses predict this orthogonality?

We examined simulated population responses from four models of motor cortex: a model that codes kinematics (coding model), a simple dynamical systems model^11^ (generator model), a recurrent neural network with non-normal dynamics^54^ (RNN1) and an RNN with regularized dynamics^42^ (RNN2). All models produced single-unit responses that resembled those of the recorded neurons. However, none of the models showed the orthogonality between the subspaces defined by the prep-PCs and move-PCs that we observed in the neural data (Fig. 4c, alignment index for the data is significantly lower than that of the models). With the exception of the coding model, the models above employ a null-space gating strategy in which preparatory activity does not influence muscle activity, because the preparatory dimensions are orthogonal to the motor-output dimensions^41^. One might initially have expected such models to exhibit little alignment, but in fact the reverse is true. As the prep-PCs are fully (generator model) or partially (RNN1 and RNN2) contained within the move-PCs, these models exhibit substantial alignment.

To ensure the robustness of these results, despite differences in the task and recording methods from previous data sets, we also analysed data sets that we previously recorded using multi-electrode arrays during a task paradigm with many conditions (different reach targets and curvature^11^; Supplementary Fig. 4). The same result holds: the prep-PCs and move-PCs were not only close to orthogonal, but were more orthogonal than expected by chance (Supplementary Fig. 4c).

### Separating preparatory and movement subspaces

The unexpected degree of orthogonality between the prep-PCs and move-PCs opens the door to new analysis approaches for understanding cortical computation. A common challenge in systems neuroscience is to independently study two related computations or representations that are both present in the same neural population. A natural approach is to segregate the population into categories, but in many brain areas neurons do not fall neatly into categories^15^,^21^,^25^,^26^. Thus, this approach will fail in these scenarios. In the present study, most neurons were active during both preparatory and movement epochs, with no evidence of discrete categories (Fig. 3c). Our results suggest an alternative approach. As preparatory and movement-related computations take place in nearly orthogonal subspaces, it should be possible to isolate those computations by projecting the full population responses onto the two subspaces, an approach similar to that in ref. 18. It is noteworthy that this separation would not be achievable in general: for example, all models in Fig. 4 involve overlapping subspaces, which would make it impossible to separate preparatory and movement-related activity via linear projections. To achieve this separation for the data, we could have projected neural responses directly onto the top prep-PCs and the top move-PCs, as they are naturally very close to orthogonal. However, the interpretation of population projections is simplest when the basis set is perfectly orthogonal. We therefore designed a dimensionality reduction method that leverages the orthogonality between preparatory and movement activities to identify two orthogonal sets of bases (Methods): one defining the preparatory subspace and the other defining the movement subspace. Using this method, we identified two preparatory dimensions (which captured 68% and 70% of the preparatory-epoch variance for monkey B and A, respectively) and four movement dimensions (which captured 66% and 73% of the movement-epoch variance for monkey B and A, respectively). Most neurons contributed to both the preparatory and movement subspaces. PMd neurons contributed slightly more to the preparatory subspace than did M1 neurons and M1 neurons contributed slightly more to the movement subspace than did PMd neurons.

**Figure 5:**
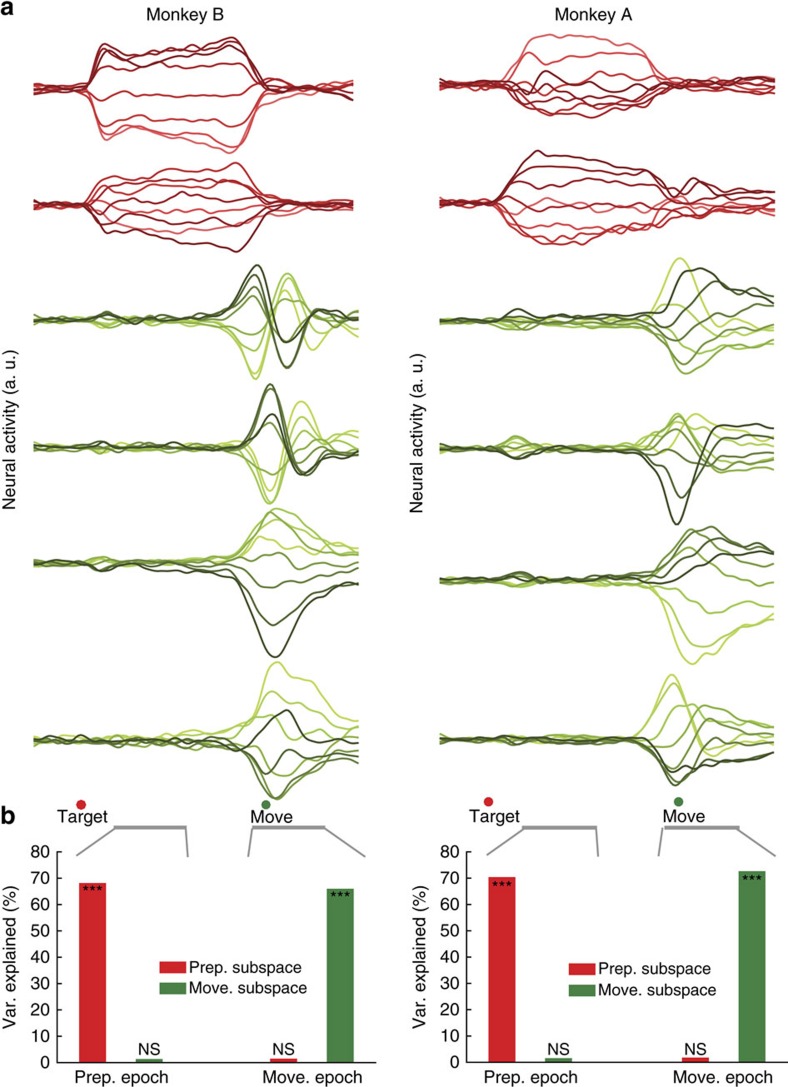
Separating preparation-related and movement-related aspects of the population response. (**a**) Projections of the neural population responses onto the two-dimensional preparatory subspace (red traces) and the four-dimensional movement subspace (green traces). Light-to-dark colour shading corresponds to different reach conditions (right-to-left). (**b**) Percentage of variance explained by the preparatory (red) and movement (green) subspaces. The left pair of bars corresponds to variance captured during the preparatory epoch. The right pair of bars corresponds to variance captured during the movement epoch. Stars denote significantly higher variance (*P*<0.001, bootstrap one tailed test) with respect to random subspaces of the same dimensionality as the preparatory and movement subspaces (NS, not significant).

We projected the population response onto the preparatory and movement subspaces. Projections onto the preparatory subspace (Fig. 5a, red) were active (the neural state varied across conditions) during the preparatory epoch but not during the movement epoch. Conversely, the projections onto the movement subspace (Fig. 5a, green) were active during the movement epoch but not during the preparatory epoch. To document the strength of this separation, we quantified the variance captured (Fig. 5b). This separation could not occur unless preparatory and movement-related activities truly existed in nearly orthogonal subspaces (Supplementary Fig. 5) and thus it confirms the results of Fig. 4. The clean separation observed in Fig. 5 was not sensitive to our choice of dimensionality; for example, the same effect was observed if we considered ten-dimensional preparatory and movement subspaces (Supplementary Fig. 6). We stress that our subspace identification method did not constrain each subspace to have low activity outside its relevant epoch. In fact, if the subspaces occupied during each epoch were not naturally close to orthogonal, the subspaces identified by our method would necessarily have been occupied during both epochs (Supplementary Fig. 5). These findings reveal that although preparatory-related and movement-related activities are mixed at the single-neuron level, they can readily be separated at the level of the population via simple linear projections onto the relevant subspaces. To explore the robustness of this separation, we also applied this method to previously published data^11^ (Supplementary Fig. 7). A clean separation of preparatory and movement-related activity was observed, and thus appears to be a very consistent feature of the motor cortex population response.

**Figure 6:**
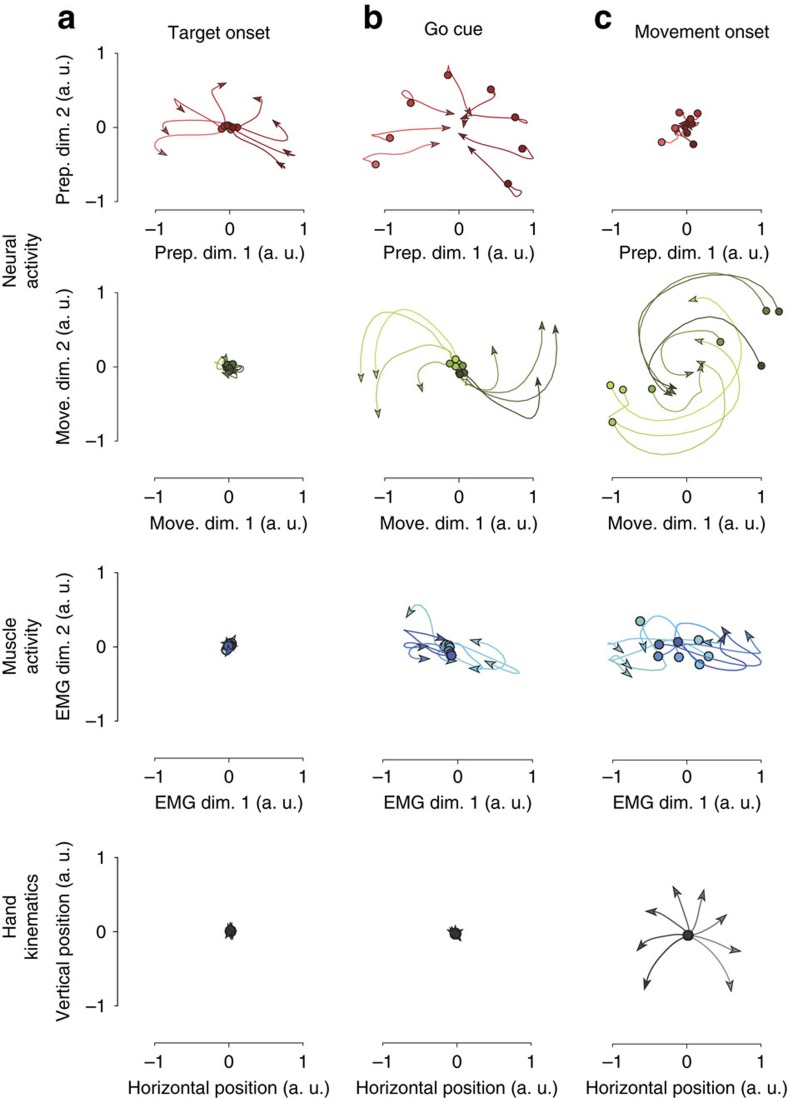
Activity in four subspaces in response to key task events. Each trace corresponds to a different reach direction. (**a**) Responses during a 150 ms window beginning at target onset. Data are shown for the neural population response (monkey B) projected onto two dimensions of the preparatory subspace (top), for the neural population response projected onto two dimensions of the movement subspace (second from top), for the top two principal components of muscle activity (second from bottom) and for hand position (bottom). (**b**) Same as in **a** but for the response to the go cue (during a 250 ms window starting at the go cue and ending at approximately the onset of movement). (**c**) Same as in **a** but for a 200 ms window starting at movement onset.

We leveraged the separability described above, to examine the evolution of the population response in state space in relation to three key events (target onset, go cue and movement onset; Fig. 6, all analyses repeated in Supplementary Fig. 8 for monkey A). During the 150 ms after target onset (that is, the early part of the delay period), neural trajectories spread out in the preparatory subspace and there was little change in activity in the movement subspace (Fig. 6a). In addition, there was essentially no change in the activity in the muscles of the arm and the monkey's hand did not move. During the 250 ms interval from the go cue time until the approximate time of movement onset, trajectories in the preparatory subspace converged; by the end of that period, there was little difference across conditions. Over the same interval of time, trajectories in the movement subspace began to spread out and the muscles started to become active (Fig. 6b). During the 200 ms after movement onset, the neural trajectories in the preparatory subspace remained converged, showing little difference across conditions, whereas the trajectories in the movement subspace exhibited strong rotational structure (Fig. 6c). During this time, there was robust muscle activity and the hand was moving.

### Link between activity in preparatory and movement subspaces

**Figure 7:**
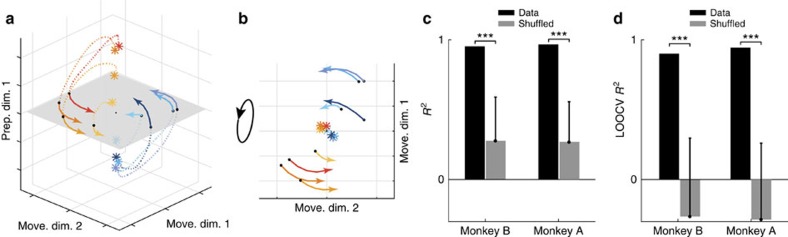
Neural activity displays an orderly transition from the preparatory subspace to the movement subspace. (**a**) Neural state trajectory during the transition from preparation to movement. Each trace plots the trajectory for one (of eight) reach directions. Axes correspond to the top preparatory dimension and the top two movement dimensions. Stars denote the neural state 200 ms before movement onset. Dotted lines denote the neural trajectories during the transition, over the next 200 ms, from the preparatory dimension to the movement dimensions. Solid lines denote the trajectories during the 50 ms following movement onset. (**b**) The same space as in **a** rotated to show only the two movement dimensions. (**c**) Quality of fit (*R*^*2*^) of the regression between the responses in the movement subspace (at the middle of movement epoch; 100 ms after movement onset) and the responses in the preparatory subspace (at the end of the preparatory epoch; 450 ms after target onset). (**d**) Leave-one-out cross-validation (LOOCV) for that same relationship. Stars in **c** and **d** denote a significantly higher *R*^*2*^ than shuffled data (*P*<0.001, one-tailed test). The shuffled data were generated by randomly shuffling the preparatory-epoch responses across conditions. In both **c** and **d**, bars show the median and error bars denote the 95% confidence interval of the shuffled distribution with 1,000 random shuffles.

The finding that preparatory and movement-related computations are performed in nearly orthogonal subspaces might initially seem contradictory in the face of previous results that preparatory and movement activity patterns are linked^3^,^6^,^32^,^33^,^34^,^35^,^36^,^37^. Yet, there is in fact no paradox: it is possible to transfer information from one subspace to an orthogonal subspace, provided the appropriate dynamics are present during the transition. This could allow motor cortex to employ a strategy similar to that outlined in Fig. 1a,d, with the relationship between conditions being conserved across epochs. To test this possibility, we examined neural trajectories during the transition from preparation to movement, plotted in the three-dimensional space spanned by the top two movement dimensions and the top preparatory dimension (Fig. 7a). These were the same dimensions as in Figs 5 and 6. Two hundred milliseconds before movement onset (stars), the neural state varied across conditions in the preparatory dimension (Fig. 7a, vertical axis), but not in the movement dimensions (stars in Fig. 7b). As time progressed, the set of neural states left the preparatory dimension (dotted traces in Fig. 7a) and entered the movement dimensions (solid traces in Fig. 7a,b). The transition from preparation to movement was not haphazard: the ordering of conditions in the preparatory dimension was closely related to the ordering of conditions in the movement dimensions.

We used linear regression to ask how well the pattern of movement activity could be predicted from the pattern of preparatory activity. We considered the two-dimensional preparatory subspace and a four-dimensional movement subspace. Subspaces were the same as in Figs 5 and 6. We used the neural state at the end of the preparatory epoch (450 ms after target onset, before the go cue) in the preparatory subspace to predict, using a simple linear decoder (Methods), the neural state at the middle of the movement epoch (100 ms after movement onset) in the movement subspace. Despite the simplicity of the decoder and the fact that preparatory and movement responses occupy orthogonal subspaces, the quality of the prediction was quite high (*R*^2^=0.95 for monkey B; *R*^2^=0.97 for monkey A; Fig. 7c). This relationship generalized well on test conditions that were not used to fit the decoder (Fig. 7d). This strong relation between the preparatory and movement subspaces persisted during the entire movement epoch; the *R*^2^ remained high even if we considered movement states at earlier or later times (data not shown). Thus, the activity pattern in the preparatory subspace is closely linked to the activity pattern in the movement subspace, consistent with previous studies^12^,^37^, even though the subspaces themselves are orthogonal.

**Figure 8:**
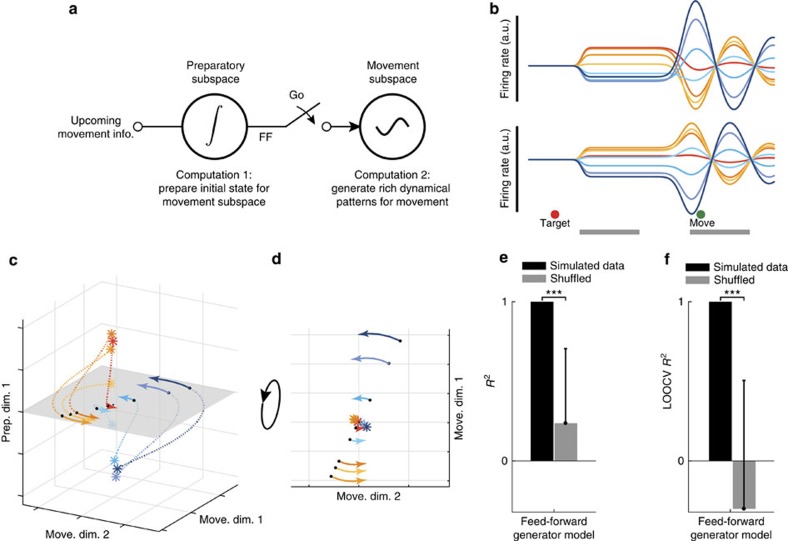
Feed-forward generator model. (**a**) Diagram illustrating the model and the computation it performs. The model consists of two latent dynamic subspaces (preparatory and movement). The movement goal (that is, target location) is loaded into the preparatory subspace. That preparatory subspace possesses leaky integrator dynamics and the input thus produces a fixed point that is specific to each condition. At the start of the movement, the state established in the preparatory subspace is passed to the movement subspace via a feed-forward mechanism. This sets the initial state in the movement subspace, whose dynamics are modelled as an oscillator. The dynamics of the preparatory and movement subspaces are fixed; different movement-subspace trajectories result from being passed different preparatory states, which in turn result from different inputs. (**b**) Responses of two simulated neurons from the model using the same conventions as Fig. 2c. (**c**) Neural trajectories from the model during the transition from preparation to movement, plotted in the top preparatory dimension and the top two movement dimensions using the same conventions as Fig. 7a. (**d**) Rotated view of **c** to show only the two movement dimensions. (**e**,**f**) Same analysis as in Fig. 7c,d but for the model data.

We constructed a highly simplified model (Fig. 8) to illustrate how some of the basic features of the data—at both the single-neuron and population level—can be reproduced by a straightforward flow of activity from one subspace to another. This model has a modular architecture related to previous work^55^, where a context switch controls the flow of information from one processing stage (the preparatory computation) to another (the movement computation). The reaching goal (that is, information about target location) serves as the input to a preparatory subspace. For simplicity, we modelled a two-dimensional preparatory subspace with simple leaky-integration dynamics: the inputs create a fixed point to which the neural state rapidly decays (one could have included additional transformations or computations, but that is unnecessary for the current illustration). Right before the movement onset time, the state is fed forward from the preparatory subspace to the movement subspace, which then engages the dynamics of the movement subspace (modelled as a two-dimensional subspace with rotational dynamics). The two computations performed by this model, although different, are linked because the preparatory state establishes the initial condition of the movement-subspace dynamical system, thus setting the amplitude and phase of the rotational trajectories during movement. Movement dynamics are initialized by a brief temporal overlap between the preparatory and movement activities, allowing a transient flow of information from the preparatory subspace to the movement subspace. The behaviour of this model is robust to the length of this brief temporal overlap, within reason: if too short, then there can be no flow of information across epochs (for example, if preparatory activity has decayed before the initiation of movement activity); if too long, preparatory activity acts as an ongoing input to the movement space, rather than acting as an initialization.

Using this simplified model, we generated simulated responses for 127 neurons (Methods). Each neuron's response was a random linear mapping of the four-dimensional (preparatory and movement) latent state space. Single neuron responses from this model show temporally complex patterns and the tuning of neurons tends to change across epochs (Fig. 8b). In addition, pairs of neurons with similar response tuning during the preparatory epoch (Fig. 8b) showed no reliable similarity during the movement epoch (*cf.* Fig. 3a,b). Because in this model preparatory and movement computations take place in different subspaces by construction, preparatory and movement subspaces are orthogonal and the alignment index is low (0.04). Furthermore, since the preparatory state is fed forward to the movement subspace (Fig. 8c,d), preparatory responses accurately predict the upcoming movement responses: the *R*^2^ between responses in the preparatory and movement subspaces was high (Fig. 8e,f), similar to the real neural data. Thus, a very simple model can capture the single-neuron and population-level features that we considered in this study.

This simple model is consistent with a basic principle embodied in previous models^11^,^12^,^42^: movement dynamics are initiated by the preparatory computation^3^. Yet, in the present model, the initiation of movement dynamics is performed indirectly through a feed-forward mechanism. The flow of information from the preparatory subspace to the movement subspace is gated by a strong nonlinear mechanism (simulated directly as an all-or-nothing switch). It remains unclear how such an effect would be mediated in a more realistic network model. In particular, the model of Sussillo *et al*.^42^ resembled the neural data precisely because regularization produced dynamics with a good linear approximation. A challenge for future work will be to build networks that retain this approximate linearity during movement, while still embodying the strong nonlinearity required to suddenly transition between the preparatory and movement subspaces.

## Discussion

We investigated three potential kinds of population structure that could occur during preparatory and movement computations. Different computations could take place (i) as independent activity patterns in orthogonal subspaces, (ii) in overlapping neural subspaces or (iii) in orthogonal*-*but-linked neural subspaces. We found that M1/PMd uses the orthogonal-but-linked strategy. The population response occupies orthogonal subspaces during preparation and movement. This finding was manifested in the large change in pairwise neuronal correlations between the preparatory and movement epochs, and in the near orthogonality between the top prep-PCs and the top move-PCs. It was therefore possible to isolate response patterns that were almost purely preparatory or purely movement related. This separation was accomplished via linear projections onto orthogonal sets of dimensions—something that is possible only when preparatory and movement subspaces are orthogonal and non-overlapping. Yet, despite orthogonality, we found that the activity pattern in the preparatory subspace accurately predicted the upcoming activity pattern in the movement subspace. In fact, it was possible to view activity flowing from the preparatory subspace into the movement subspace just before movement onset. Critically, this flow largely preserved the relationship between conditions. Although the possibility that motor cortex uses this population-level strategy had not previously been considered, these results agree with the longstanding hypothesis that there exists a lawful relationship between preparatory and movement computations^5^,^6^,^12^,^32^,^33^,^34^,^35^,^36^,^37^,^56^.

A corollary of orthogonality between the preparatory and movement-related subspaces is that the same population shows fundamentally different properties during the two epochs. In particular, during preparation there exist sets of neurons that share similar tuning. It is common to interpret similarity of tuning as indicating a basic cooperation between neurons—two neurons with similar tuning perform a similar function. Yet, here the nature of that cooperation changes completely in only a few tens of milliseconds. Two neurons that appeared to be cooperative during the preparatory epoch show almost no tendency to be similarly cooperative during the movement epoch. Thus, cooperation between neurons may not be a fixed property of the circuit, but can change rapidly as the underlying computation changes^22^,^57^. This finding carries implications regarding how data should be interpreted and analysed. For example, basing an analysis or readout on the pattern of responses during one epoch may fail to yield interpretable results when applied to another epoch. Yet, orthogonality also allows analysis opportunities that would not otherwise be available: in the present case the ability to separate activity related to preparation from activity related to movement, allowing independent study of each computation.

How might two computations that occur in orthogonal neural subspaces be linked? A potential answer to this question comes from neural network theory, where the linkage could be achieved by a feed-forward mechanism from one subspace to the other^58^,^59^. We implemented a simplified model of this strategy. This model produced structure that agrees with the orthogonal-but-linked structure that we observed in the motor cortex data: the preparatory state determined the subsequent phase and amplitude of movement-period oscillatory activity, but only after a flow of activity from the preparatory subspace into the movement subspace. This model is not a neural network and is intentionally highly simplified, yet it exhibits a key feature (orthogonality of subspaces) that is not present in any other model, including a recent dynamics-based model of activity in motor cortex^11^ and large-scale network models^42^,^54^.

The reason that prior conceptual and network models do not account for the present results is that in those models the preparatory subspace overlaps with (is largely or fully contained within) the movement subspace. The movement subspace contains both output-potent dimensions that directly influence the muscles and many output-null dimensions that do not. Preparatory activity in those models occupies only the output-null dimensions, but is still contained within the movement subspace. Thus, prior models assume the overlapping strategy. The overlapping strategy emerges during model optimization and is a natural means for linking preparatory and movement computations, while preventing preparatory activity from directly causing motor output. Here it is worth stressing a subtle but important point: prior models that exploit an output-null space do not reproduce the large degree of orthogonality between prep-PCs and move-PCs nor was that degree of orthogonality anticipated in the context of empirical evidence, presented by Kaufman *et al*.^41^ that motor cortex leverages an output-null space (Supplementary Fig. 2). Nevertheless, our present findings are consistent with the central message of Kaufman *et al*.^41^ that motor cortex leverages a null space to avoid causing movement during preparation. Indeed, the orthogonality observed here necessarily implies (but is not implied by) the null-space result of that work; if preparatory dimensions are orthogonal to the full set of movement dimensions, then they are necessarily orthogonal to the subset of movement dimensions that drive muscle activity. We have also re-analysed the data sets used in Kaufman *et al*.^41^ using our current approach and confirmed that the present results hold.

Although previous models fail to reproduce our current finding, they successfully reproduce many previously known features of motor cortex responses, including the rotational structure present during movement^11^. In particular, the model of Sussillo *et al*.^42^ showed qualitative and quantitative similarity to the empirical data in a variety of ways. A future goal will be to explore how modifications of such models might allow them to also account for the present result. Although these modifications should be possible, they are unlikely to be trivial: in our simple model the sudden transition from the preparatory to movement subspaces was achieved via an explicit gate, and although networks could probably achieve the same effect using strong nonlinearities, it is not yet clear how this could be accomplished while also maintaining a key point of agreement with the empirical data: the approximate linearity of dynamics during the movement period. Our results thus provide a challenge to neural network theory to explain how a single population can completely change its subspace and dynamics between computations, while still showing smooth and well-behaved dynamics within a computation.

It is intriguing that motor cortex uses the orthogonal-but-linked strategy, given that network models are capable of successfully producing muscle activity using the overlapping strategy. Intuitively, the orthogonal-but-linked strategy may provide greater flexibility or better learnability. Different subspaces may possess different dynamics suitable for different computations, including not only the computations observed within our task but other computations necessary for other classes of movement. One possibility suggested by theoretical neuroscience is that large RNNs possess reservoirs of component response patterns that can be combined to perform particular computations^60^,^61^,^62^. Each reservoir component corresponds to a dimension in the high-dimensional neural space. Different computations can be performed by recruiting (via inputs or via training) different (and thus orthogonal) components. This suggestion is necessarily speculative, but such a strategy is plausible and would naturally lead to different computations occupying orthogonal subspaces. A prediction of this hypothesis is that orthogonality should be observed whenever two computations require different components within the larger reservoir. This could lead to the orthogonal-but-linked strategy when successive computations must influence one another and the independent strategy when they should not. For example, Kurtzer *et al*.^20^ found random changes in single-neuron tuning gain between posture and movement. Their interpretation—that different control processes are at play during posture and movement—accords with the above interpretation. Postural and movement control involve very different timescales and the appropriate components of a reservoir would therefore probably differ. Still, it should be stressed that it may often be difficult to intuit, a priori, when two contexts require different computations.

A natural consequence of a change in subspace from one epoch to the next is complex and seemingly arbitrary changes in neural tuning (Supplementary Note 1). For example, a neuron that responded most vigorously during preparation of a rightwards movement may respond most vigorously during execution of a leftwards movement, or may not respond at all during movement. Such response complexity is commonly observed across the brain^16^,^17^,^18^, especially in frontal areas^13^,^14^,^63^,^64^, raising the possibility that the orthogonal-but-linked strategy may be used outside of motor cortex when two related computations are performed by the same population. Indeed, recent results in rodent parietal cortex during a multi-sensory decision task are consistent with the orthogonal-but-linked strategy^21^. That study found that neural activity in posterior parietal cortex during a decision (based on auditory and visual stimuli) was only weakly captured by the neural subspace occupied during the subsequent orienting movement towards the selected reward port. Despite such orthogonality, it is likely that decision-related and movement-related activities are linked. In general, explanations couched in terms of linked computations in orthogonal subspaces could be a more useful account of response complexity than the surface-level fact that tuning (or the neural code) changes^26^,^27^,^29^,^65^,^66^. However, we stress that surface-level complexity does not imply the presence of orthogonal subspaces. Indeed, one cannot distinguish between the three scenarios in Fig. 1 at the single-neuron level: all may lead to complex responses with seemingly arbitrary changes in tuning (Fig. 1e–g and Supplementary Note 1). Fortunately, the conceptual and methodological approach introduced here can be readily applied to population responses from almost any brain area and will reveal which population-level response structure is responsible for the observed single-neuron response complexity.

## Methods

### Subjects

We trained two male rhesus macaque monkeys (*Macaca mulatta*) aged 8 and 12 years, and weighing 11–13 kg. Monkeys were trained to reach for targets to obtain juice reward. All procedures were in accordance with the guidelines of the US National Institutes of Health and were approved by the Columbia University Institutional Animal Use and Care Committee.

### Task

Subjects sat in a chair facing an LCD display and reached with their right arm. We tracked hand position using an infrared optical system to track a reflective bead temporarily affixed to the third and fourth digits. Each trial began when the monkey touched and held a central touch point for 450–550 ms (randomized). A small target then appeared in one of eight possible locations radially arranged around the central touch point at a distance of 130 mm. Following a variable (0–1,000 ms) delay, reach initiation was instructed by a go cue. For some trials the go cue was a change in the size of the target, whereas for other trials the go cue was a radial change in target position. The purpose of these two go cues was incidental to the present study and data were averaged across all trials regardless of the identity of the go cue. Monkeys were required to begin the reach with reaction times between 100 and 500 ms, and to hold the target for 600 ms to receive a juice reward.

### Neural and EMG recording

After subjects became proficient in the task, we performed sterile surgery to implant a head restraint. At the same time we implanted a standard recording chamber centred over the hand and arm area of the primary motor cortex (M1) and the dorsal aspect of premotor cortex (PMd) of the left hemisphere. Chamber positioning was guided by structural magnetic resonance images taken shortly before implantation. We used intracortical microstimulation to confirm that our recordings were from the forelimb region of motor cortex. Microstimulation typically evoked contractions of the shoulder and upper-arm muscles, at currents from 5 to 60 μA depending on the location and cortical layer. We recorded single-neuron responses using a tungsten electrode (FHC) lowered into cortex using a motorized microdrive. Spikes were sorted online using a window discriminator (Blackrock Microsystems). We recorded all well-isolated task-responsive neurons and no attempt was made to screen for neuronal tuning for reach direction or any other response property. Spikes were smoothed with a Gaussian kernel with s.d. of 20 ms and averaged across trials to produce peri-stimulus time histograms. Because the time between target onset and movement onset was variable, for each condition we produced two averages: one time-locked to each event. These averages were spliced to yield a single firing rate trace as a function of time for each reach condition.

For both monkeys, we recorded electromyogram (EMG) activity using intramuscular electrodes from the following muscles: lower and upper aspects of the trapezius, medial, lateral and anterior aspects of the deltoid, medial and outer aspects of the biceps, brachialis, pectoralis and latismus dorsi. EMG signals were bandpass filtered (10–500 Hz), digitized at 1 kHz, rectified, smoothed with a Gaussian kernel with standard deviation of 20 ms, and averaged across trials to produce peri-stimulus time histograms. The neural and EMG responses were then sampled every 10 ms.

### Data preprocessing

For analyses based on correlation, PCA, subspace identification and relationship between preparation and movement (that is, all figure panels except Figs 1, 2a,b, 3c and 8), we followed the two pre-processing steps used in our previous work^11^. First, neural responses for each neuron were soft-normalized such that neurons with strong responses had approximately unity firing rate range (normalization factor=firing rate range+5). Second, the neural responses for each neuron were mean-centred at each time as follows: we calculated the mean activity across all conditions of each neuron at each time point and subtracted this mean activity from each condition's response. These two preprocessing steps were also applied to EMG and simulated neural data.

In terms of outliers, there were two neurons from monkey A (none from monkey B) that were active exclusively during the movement epoch (that is, their firing rates were zero during the preparatory epoch). As correlation is not defined when a neuron has exactly zero firing rate, these two neurons were excluded from all analyses, except the epoch-preference analysis below (where their inclusion is essential to avoid biasing the result).

### Epoch-preference index

We measured the strength of tuning of neuron *i* separately for the preparatory and movement epochs by calculating the maximum range of the neuron's firing rate (across all reach directions) in that epoch divided by the average firing rate range of neuron *i* across all times; we denote the preparatory tuning strength and the movement tuning strength as *S*_prep_(*i*) and *S*_move_(*i*), respectively. To account for the fact that preparatory and movement activity may have different average magnitudes (for example, movement activity tends to be stronger on average), we normalized the tuning of neuron *i* by the mean tuning across all neurons (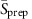
or 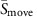). This normalization ensured that an index of zero corresponds to a neuron that showed the average ratio of preparatory-epoch tuning and movement-epoch tuning. The epoch preference index of neural activity for neuron *i* was then:


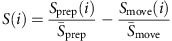


This index measures the preferential activity of a given neuron *i* during the preparatory epoch versus the movement epoch. If the distribution of this index is significantly bimodal, this implies that there exists one subpopulation of neurons that is more selective during the preparatory epoch than during the movement epoch, and another subpopulation that is more selective during the movement epoch than during the preparatory epoch.

### Pairwise cross-condition correlation

To calculate the cross-condition correlation between all neurons during the preparatory epoch, we grouped neural responses into the matrix 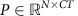, where *N* is the total number of neurons, *C* is the number of conditions and *T* is the number of time points (all times within the preparatory epoch). Similarly, we grouped the neural responses during the movement epoch into the matrix 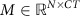 (all times within the movement epoch). The preparatory-epoch correlation matrix was obtained by calculating the correlation between the rows of the matrix *P* and the movement-epoch correlation matrix was computed analogously based on *M*.

### Subspace overlap analysis

We performed PCA on the matrix *P*, treating each row (that is, each neuron) as a variable, to obtain the prep-PCs (ten PCs). Each PC is thus a direction in *N*-dimensional neural space. Similarly, we obtained the move-PCs (ten PCs) by performing PCA on the matrix *M*. In Fig. 4a, we projected the preparatory-epoch activity (*P*) onto the prep-PCs and quantified the percent of variance explained relative to the total variance of *P* (red bars). In Fig. 4a, we also projected the movement activity (*M*) onto the prep-PCs and quantified the percent of variance explained relative to the total variance of *M* (green bars). This procedure reveals the amount of preparatory-epoch and movement-epoch variance shared in the prep-PCs. The same procedure was repeated for the move-PCs (Fig. 4b). To quantify the amount of variance shared between the preparatory and movement population responses, in Fig. 4c we projected the preparatory-epoch activity onto the move-PCs (red bars, Fig. 4b) and calculated the sum of the variance captured. We did the same projection onto the prep-PCs (red bars, Fig. 4a) and calculated the sum of the variance captured in that space. We then defined the alignment index *A* as the ratio of this sum, which can be written concisely as:


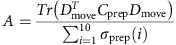


where *D*_move_ is the matrix defined by the top ten move-PCs. *C*_prep_ is the covariance of the matrix *P*. 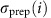 is the *i*^*th*^ singular value of 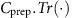 is the matrix trace. The numerator measures the amount of the preparatory-epoch data variance captured by the top ten move-PCs. The denominator normalizes the alignment index by the highest amount of the preparatory-epoch data variance that can be captured by a ten-dimensional subspace (that is, the preparatory-epoch data variance captured by top ten prep-PCs); thus, the alignment index ranges from 0 to 1.

### Identifying preparatory and movement subspaces

Here we aim to identify two mutually orthogonal bases, where one captures the preparatory activity, and the other captures the movement activity. Traditional methods could perhaps be modified to identify two orthogonal neural subspaces^13^,^41^, although this type of heuristic orthogonalization is known to be suboptimal^67^. For example, PCA can identify one subspace (for example, move-PCs) and then may be reapplied on the null space of that subspace to identify the second orthogonal subspace. This heuristic orthogonalization technique usually implies arbitrary prioritization of one subspace to be better identified (in this example the movement activity) over the other (in this example, the preparatory activity). Thus, such methods are greedy and are often sensitive to the relative amount of activity variance of the two neural population responses and their dimensionalities.

Instead, to identify the optimal preparatory and movement subspaces, we designed a method that maximizes the sum of the variance of the preparatory-epoch responses in the preparatory subspace and the variance of the movement-epoch responses in the movement subspace. Specifically, we identified the preparatory and movement subspaces by optimizing the following objective:


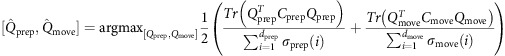



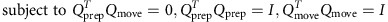


where *C*_prep_ and *C*_move_ are the covariance matrices of the neural activity during the preparatory epoch and movement epochs, respectively. 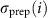 is the *i*^th^ singular value of *C*_prep_ and 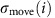 is the *i*^th^ singular value of *C*_move_. *Q*_prep_ and 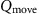 are the bases for the identified preparatory and movement subspaces, respectively. We chose the dimensionality of *Q*_prep_ to match the number of prep-PCs that capture ∼70% of variance (*d*_prep_=2) and we chose the dimensionality of *Q*_move_ to match the number of move-PCs that captured ∼70% of movement variance (*d*_move_=4). This choice did not affect the results (Supplementary Fig. 6). The term 
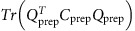
 represents the preparatory-epoch data variance captured by the preparatory subspace and 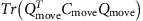 reflects the movement-epoch data variance captured by the movement subspace. Computationally, we employed recent dimensionality reduction optimization tools^67^,^68^. This method is novel in that it simultaneously identifies the preparatory and movement subspaces, while constraining them to be completely orthogonal (unlike the preparatory and movement PCs, which are largely but not completely orthogonal). In addition, the optimization objective is suitably normalized (the singular values in the above objective) to be insensitive to the relative dimensionality and amount of response variance in the two subspaces. This normalization is particularly important in our case, as the movement activity is stronger and typically has higher dimensionality than the preparatory activity. It is noteworthy that this method does not require an initial denoising step as preprocessing and does not require prioritizing one subspace to be better identified over the other. In Figs 5a, 6, and 7a,b and Supplementary Fig. 8, the identified movement subspaces were further rotated using the jPCA method^11^ to illustrate the movement-related oscillatory activity patterns.

### Relationship between preparation and movement activities

To investigate the relation between neural activity in the preparatory subspace and movement subspace, we projected the population activity for all *C* reaching conditions onto the preparatory subspace and similarly projected the movement activity onto the orthogonal movement subspace. 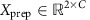 is the matrix that contains the activity projected onto the preparatory subspace at the end of the preparatory epoch (450 ms after target onset). 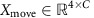 is the matrix that contains the activity projected onto the movement subspace at the middle of the movement epoch (100 ms after movement onset). To investigate the relation between *X*_prep_ and *X*_move_, we fit the following linear decoder: 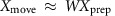 with least squares; thus 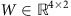 is a matrix of regression weights that links the preparatory and movement responses. We quantified the quality of the fit of this decoder by calculating an 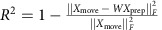. We also quantified the generalization performance of this decoder by performing leave-one-out cross-validation and measuring *R*^2^ for test conditions that were not used to fit the decoder. The data *R*^2^ was compared with a control distribution of *R*^2^-values obtained by shuffling each row of *X*_prep_ independently and fitting the same linear decoder to predict *X*_move_.

### Simulated data

Simulated neural responses (Fig. 4c) were generated using four previously published models. A full description of each is in Supplementary Note 2; here we briefly describe the critical choices. The first model is a coding model. Neural responses from this model were a linear function of different kinematic parameters. The responses during movement were related to the hand position, velocity, acceleration and jerk, whereas during preparation the responses were related to the reach end points and maximum reach speed. The second model is a pattern generator model^11^. A dynamical oscillator generates the simulated responses during movement with oscillation phase and amplitude determined by the preparatory activity. The model was optimized to produce the empirical patterns of muscle activity. The third model (RNN1) is a non-normal^58^ RNN^54^. The preparatory responses are simulated by the input phase that holds the network at an initial fixed point. The movement responses are the dynamical evolution of the network from this initial point. The network units are constrained such that units can be either only excitatory or only inhibitory. The fourth model (RNN2) is a RNN presented in detail in Sussillo *et al*.^42^ Similar to RNN1, the simulated preparatory neural responses in RNN2 are related to the input phase and the movement neural responses are related to the dynamical evolution phase. However, unlike RNN1, RNN2 units are randomly initialized and the network was explicitly optimized to produce the empirical patterns of muscle activity, with strong regularization encouraging smooth dynamics.

We also simulated a simple model to illustrate how activity can flow from one subspace to an orthogonal subspace (feed-forward generator model). The responses of this feed-forward generator model at different times (*t*) and different conditions (*c*) were generated from the following dynamical system:


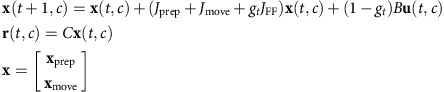


where **x**_prep_ and **x**_move_ are the preparatory and movement latent variables, respectively. *J*_prep_ is the dynamics matrix of the preparatory subspace (a leaky integrator) and *J*_move_ is the dynamics matrix of the movement subspace (an oscillatory pattern generator). *J*_FF_ is the matrix describing the feed-forward dynamics from **x**_prep_ 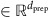
to 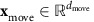. We chose the dimensionality of **x**_prep_and **x**_move_ to be equal to 2 (*d*_prep_=2 and *d*_move_=2). The feed-forward dynamics are gated by the go signal *g*_*t*_, which remains 0 at all times before the go cue and smoothly reaches a maximum of 1 right before the movement time (step function smoothed with a 20 ms s.d. Gaussian kernel). *B* is the loading matrix that loads the input (**u**) to the dynamical system; *B* is chosen such that the only **x**_prep_ is loaded with the input. For each condition *c*, the input **u** smoothly changes from 0 to a value u*(*c*) right after the target onset time and remains constant afterwards. u*(*c*) is chosen as the reach target position of condition *c*. The input is gated by (1−*g*_*t*_), which deactivates the input during the movement epoch when preparatory computation is no longer needed.*C* is a loading matrix that defines the relation between the simulated firing rates vector of all neurons (**r**) and the vector of dynamic latent variables (**x**). For the simulated data in Fig. 8, we used the following parameters for the model:


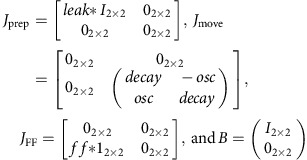


We set parameters as follows: 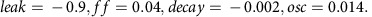 *I*_2 × 2_ is the identity matrix of size 2 by 2, 0_2 × 2_ is the zeros matrix and 1_2 × 2_ is the ones matrix. The entries of the loading matrix 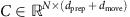 were chosen randomly from a Gaussian distribution with zero mean and s.d. of 50 (*N*=127).

### Random subspaces

One reasonable explanation of the orthogonality between the preparatory and movement subspaces is that it is expected from the fact that neural responses evolve in a high-dimensional neural space (one dimension per neuron). Do random data—data that share the neural covariance *C* with the real data, but are otherwise random—show this orthogonality? We estimated the neural covariance *C* from the real neural responses obtained from data at all the times of the task. We developed a Monte Carlo analysis that sampled random subspaces (10,000 samples) in the neural space according to *C* (Supplementary Note 3). We used these random samples to calculate the distribution of alignment indices (Fig. 4c). This distribution reflects our baseline expectation for the value of the alignment index based only on the dimensionality of motor cortex data across all times.

### Data availability

The data sets generated during and/or analysed during the current study are available from the authors on reasonable request.

### Code availability

Code packages for testing orthogonality of subspaces and dimensionality reduction method for identifying orthogonal subspaces are available from the authors.

## Additional information

**How to cite this article:** Elsayed, G. F. *et al*. Reorganization between preparatory and movement population responses in motor cortex. *Nat. Commun.* **7,** 13239 doi: 10.1038/ncomms13239 (2016).

## Supplementary Material

Supplementary Information Supplementary Figures 1-8, Supplementary Notes 1-3 and Supplementary References
