# Supplementary material for: Reorganization between preparatory and movement population responses in motor cortex
Source: Nat Commun. 2016 Oct 27;7:13239. doi: 10.1038/ncomms13239 (PMC5095296; doi:10.1038/ncomms13239)
Supplement: Supplementary Information — Supplementary Figures 1-8, Supplementary Notes 1-3 and Supplementary References [file ncomms13239-s1.pdf]

Supplementary Figures

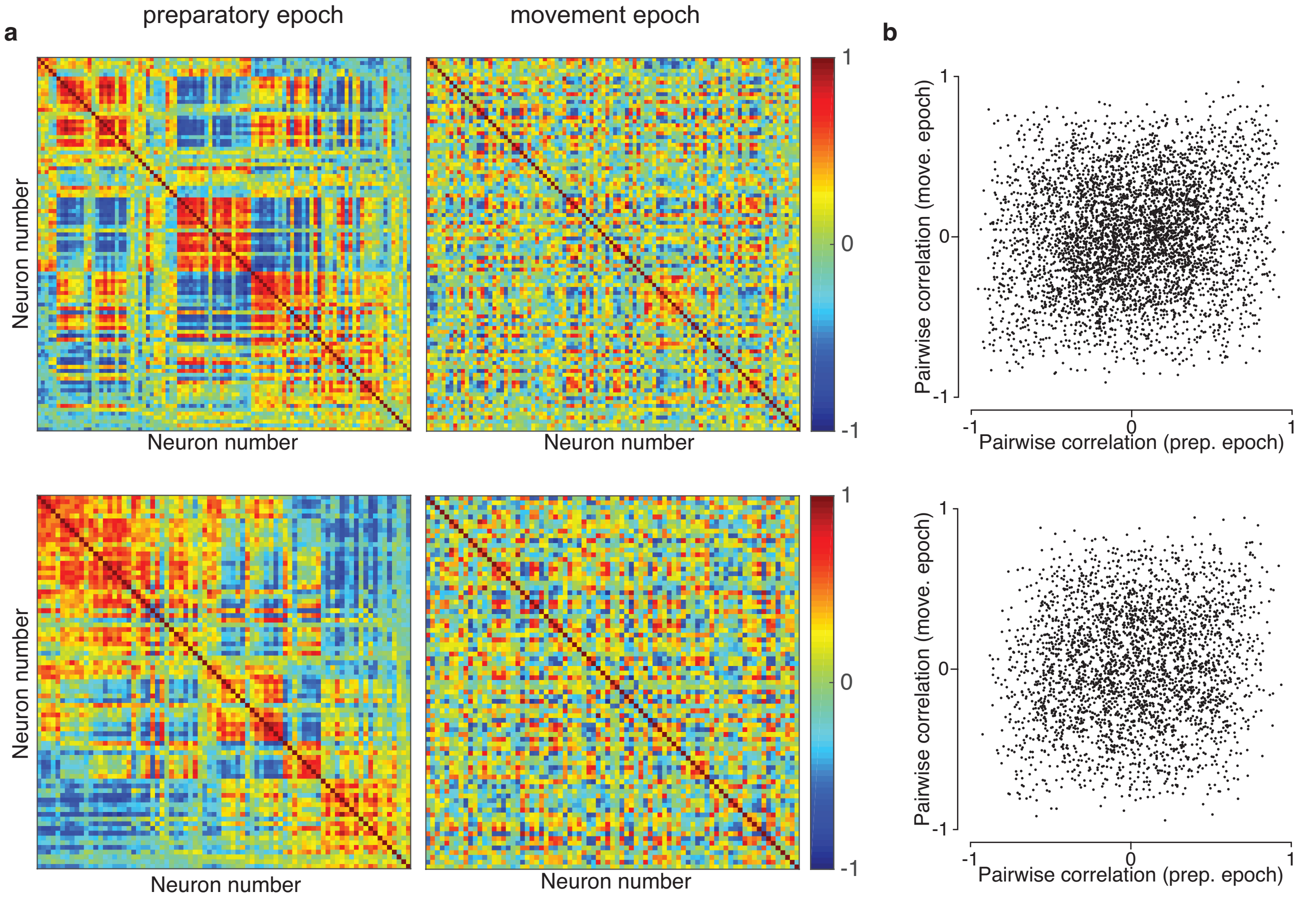

Supplementary Figure 1. Preparatory-epoch and movement-epoch correlation structure for strongly selective neurons.

This figure shows the same result as **Figure 3** from the main text but on the subset of neurons that are strongly selective at both the preparatory and movement epochs (96 neurons monkey B, 79 neurons monkey A). We considered a neuron strongly selective during the preparatory epoch if its maximum firing rate range across conditions during the preparatory epoch exceeded 8 spikes per sec, and we considered a neuron strongly selective during the movement epoch if its maximum firing rate range across conditions during the movement epoch exceeded 10 spikes per sec. Additionally, we required the maximum firing rate range for both the preparatory and movement responses to be greater than 1.5 times the firing rate range of the neuron baseline activity (activity prior to target onset). (**a**) Preparatory-epoch (left) and movement-epoch (right) correlation matrices for neurons with strong selectivity during both the preparatory and movement epochs for monkey B (top) and monkey A (bottom). Each entry in the matrix gives the degree to which the response pattern was similar for the two neurons during that epoch. The order of neurons is the same for the preparatory-epoch matrix and the movement-epoch matrix. (**b**) The correlation for each neuron pair during the movement epoch plotted against the correlation for the same pair during the preparatory epoch (R^2 = 0.13 ± 0.03 for monkey B, R^2 = 0.10 ± 0.03 for monkey A).

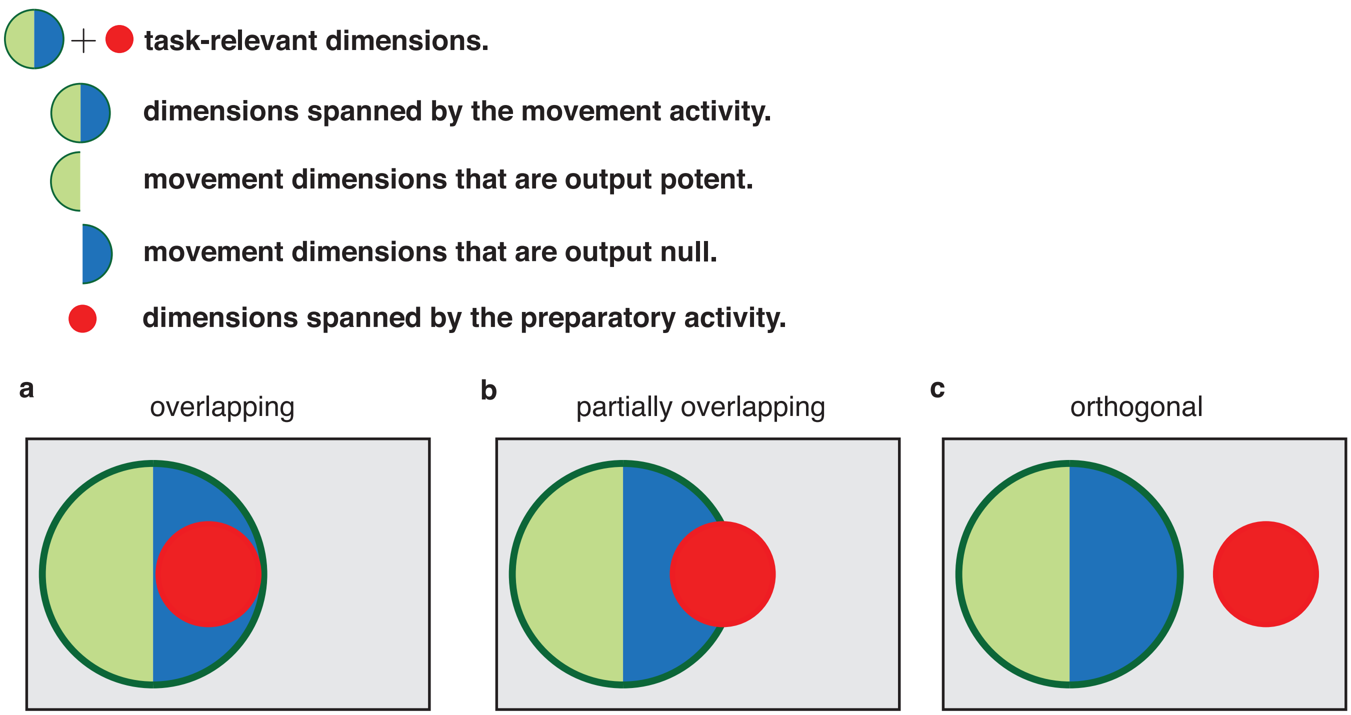

Supplementary Figure 2. Venn diagram of possible population structures that are consistent with the findings from Kaufman *et al.*[1].

That work found that preparatory activity avoids output-potent dimensions. The dimensions spanned by the movement activity (large circle) can be decomposed to dimensions that directly influence muscle (output potent; light green) and dimensions that do not (output null; blue). Kaufman *et al.* showed that the dimensions spanned by preparatory activity (red) are not contained within output-potent dimensions. Gray represents other neural dimensions not relevant to the task. (**a**) The preparatory dimensions do not overlap with the output-potent dimensions yet are fully contained (overlapping) within other (output-null) movement dimensions. This structure is consistent with the generator model of Churchland *et al.*[2], and the findings of Kaufman *et al*. (see Figures 2 and 3b in that paper). (**b**) The preparatory dimensions are partially overlapping with the movement dimensions. (**c**) The preparatory dimensions do not overlap with (are orthogonal to) the movement dimensions.

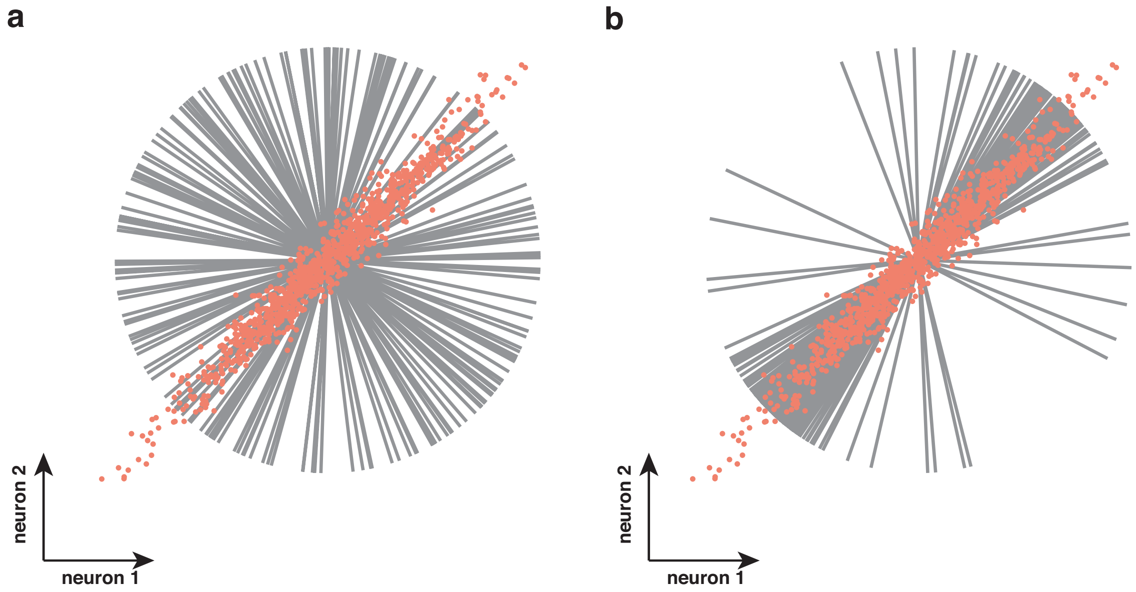

Supplementary Figure 3. Random dimensions within data space.
Two toy examples: one demonstrating uniformly sampling random dimensions in the neural space, and the other demonstrating sampling dimensions confined to the space occupied by the data. The dots are simulated responses. (**a**) Sampling random dimensions (200 gray lines). Note, the random dimensions uniformly fill the whole neural space (2D space), but are not biased to the space occupied by the data (*i.e.,* do not follow the data correlation structure). (**b**) Data-correlation aligned sampling. Note that the random dimensions (200 gray lines) are more numerous in directions more heavily occupied by the data, and thus the sampled random dimensions follow the data correlation structure.

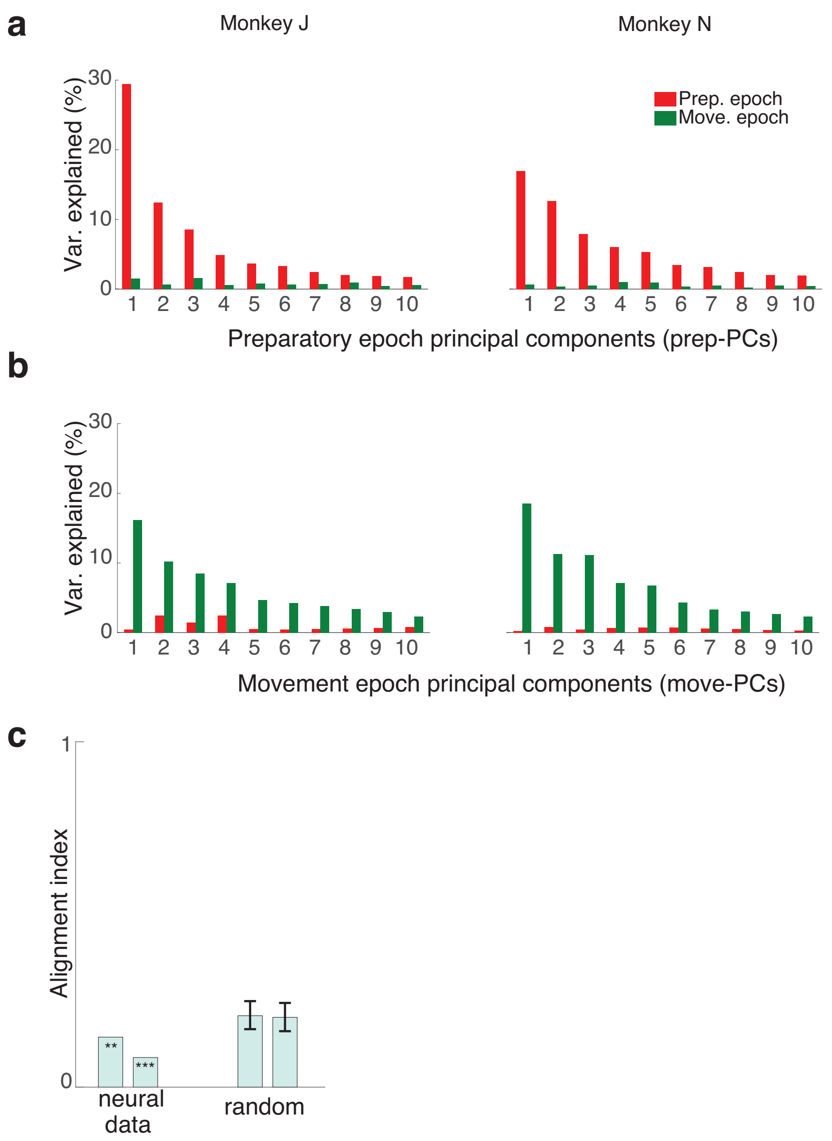

Supplementary Figure 4. Percentage of variance explained by preparatory and movement principal components (prep-PCs and move PCs).

This figure shows the same result as **Figure 4** from the main text but for multielectrode array datasets in a more complex task structure. These data are precisely those analyzed in both Churchland *et al.*[2] and Kaufman *et al.*[1] (see those papers for task description), providing a key connection to those works. (**a**) Percentage of preparatory-epoch data variance (red bars) and movement-epoch data variance (green bars) explained by the top ten prep-PCs. (**b**) Percentage of preparatory-epoch data variance and movement-epoch data variance explained by the top ten move-PCs. (**c**) Alignment index for neural and random data. For each pair the two bars correspond to data from monkey J and N, or to simulated datasets based on the real data from those two monkeys. Bars labeled 'random' correspond to the distribution of the indices expected from random dimensions within the data space. Stars for the neural data bars denote a significantly lower index relative to both random and to all models (monkey J: $p<0.01$, and monkey N: $p<0.001$; one-tailed test). For random data, the bars show the median index across multiple bootstrap resamples, and error bars denote the 95% confidence interval (based on the distribution obtained via bootstrap).

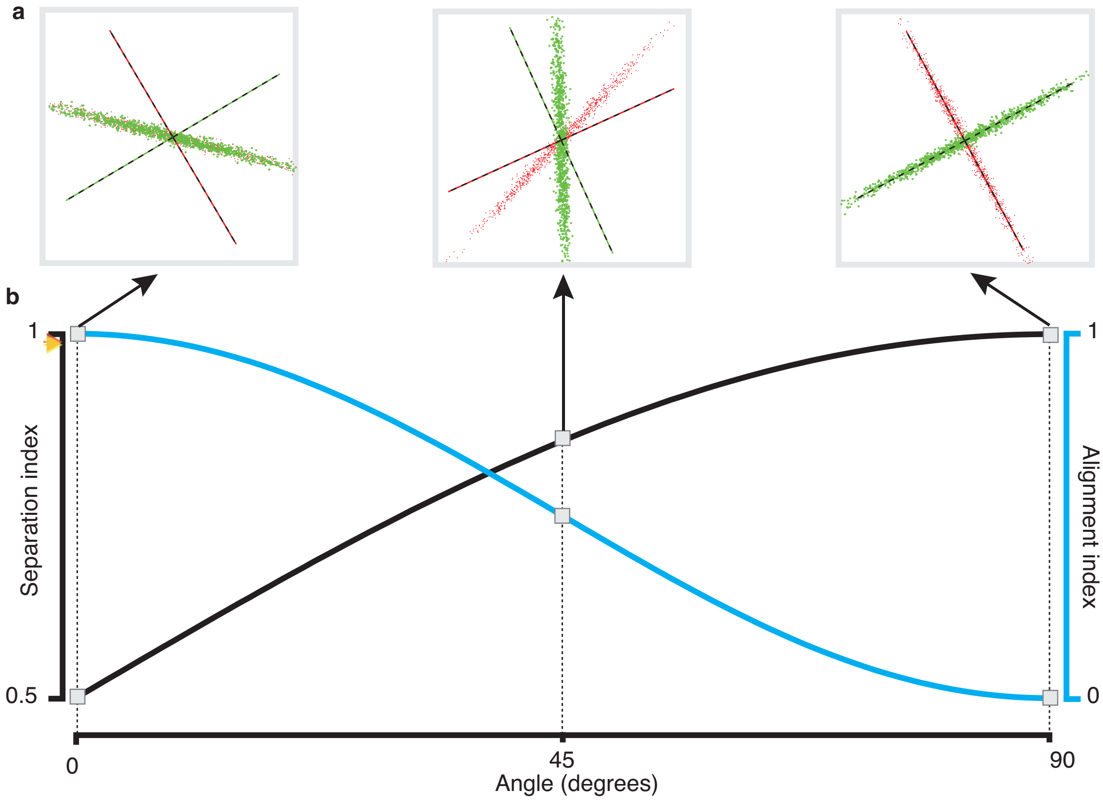

Supplementary Figure 5. Illustration of the dimensionality reduction method.
(**a**) Three examples illustrating the identification of two orthogonal subspaces using the dimensionality reduction method developed in this work when the true subspaces are completely aligned (left), partially aligned (middle), and completely orthogonal (right). The green (heavy) dots are simulated data points belonging to one epoch and the red (light) dots are simulated data belonging to another epoch. Dashed lines show the subspaces identified by the method. Note the importance of the orthogonality between the subspaces: when the true subspaces of data at different epochs are completely orthogonal (right), the identified orthogonal subspaces are identical to the true subspaces. Thus, each subspace is occupied only during its epoch, and accordingly each subspace must have high variance during its relevant epoch, and low variance during the other epoch (as shown in **Figure 5b**). This fact is reflected by the high separation index between the data at the two epochs shown in the left axis of panel **b** (this figure). Note also that if the true subspaces are completely aligned (left) or somewhat aligned (middle), the identified subspaces would be occupied during both epochs (this fact is reflected by the low separation index between the data across the two epochs). (**b**) The separation index of the responses in the identified subspaces (left axis, black). The horizontal axis denotes the angle between the subspaces in the simulated datasets (averaged over 100 simulated datasets). This index is the objective function that the dimensionality reduction method maximizes (Methods). The separation index is maximal (equaling 1) when the data across the two epochs are completely orthogonal. Note the high separation index (near 1) of the real data from monkey B and monkey A, indicated by the red and yellow arrows on the separation index axis, respectively. In blue (right axis), the alignment index (refer to **Figure 4c**) of the simulated data at the two epochs as a function of the true relation between the two subspaces (the black and blue traces are the average over 100 simulated datasets).

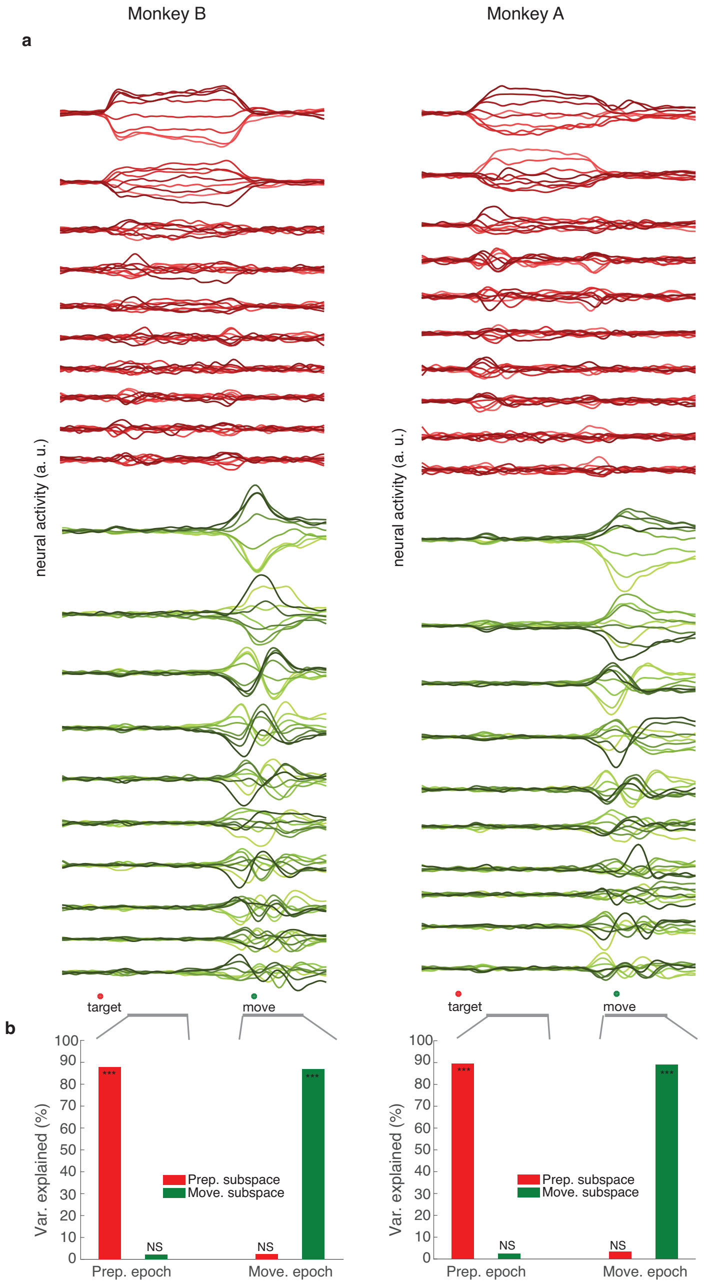

Supplementary Figure 6. Separating preparation-related and movement-related aspects of the population response.
This figure shows the same result as **Figure 5** from the main text but for a different choice of dimensionality for the preparatory and movement subspaces.
(**a**) Projections of the neural population responses onto the 10-dimensional preparatory subspace (red traces) and the 10-dimensional movement subspace (green traces). Light-to-dark color shading corresponds to different reach conditions (right-to-left). (**b**) Percentage of variance explained by the preparatory (red) and movement (green) subspaces. The left pair of bars corresponds to variance captured during the preparatory epoch. The right pair of bars corresponds to variance captured during the movement epoch. Stars denote significantly higher variance (p<0.001, one-tailed test) with respect to 10-dimensional random subspaces (NS: not significant).

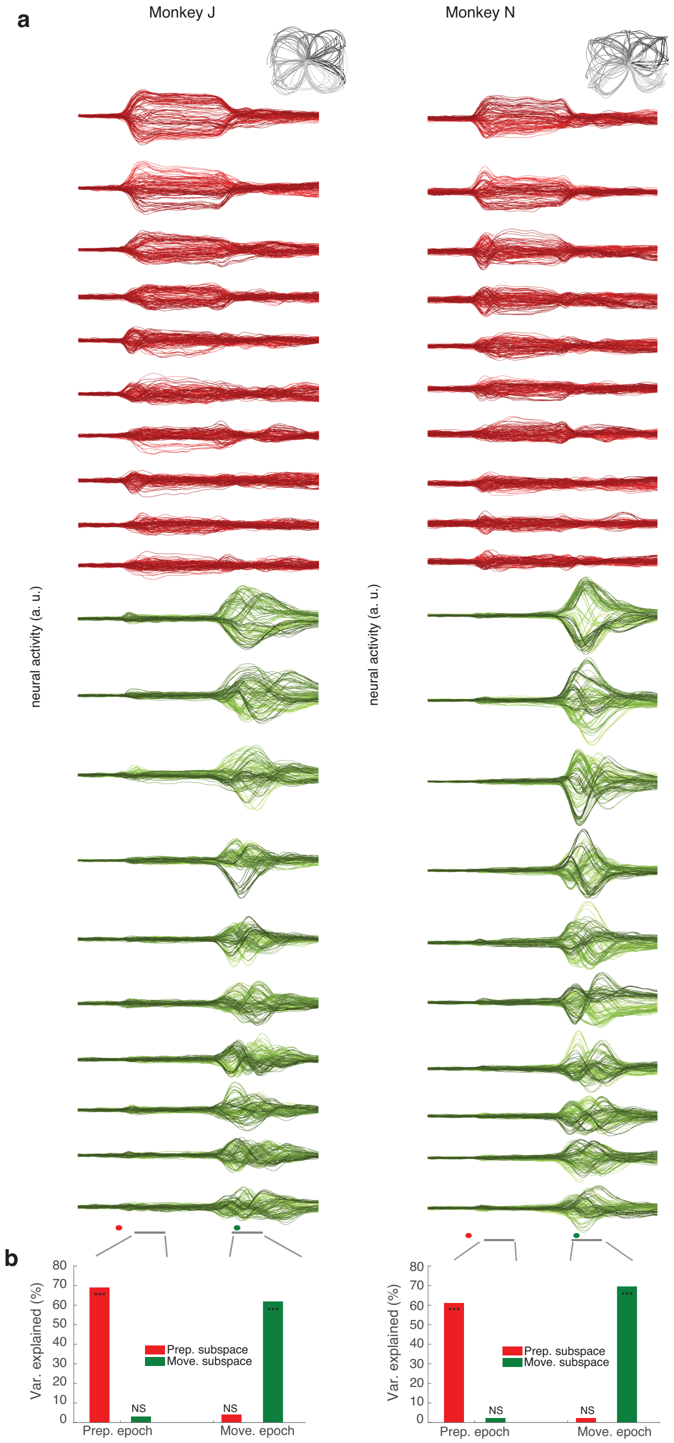

Supplementary Figure 7. Separating preparation-related and movement-related aspects of the population response (multielectrode array dataset).

This figure shows the same result as **Supplementary Figure 5** but for a multielectrode array dataset analyzed in both Churchland *et al.*[2] and Kaufman *et al.*[1] (**a**) Projections of the neural population responses onto the 10-dimensional preparatory subspace (red traces) and the 10-dimensional movement subspace (green traces). Light-to-dark color shading corresponds to different reach conditions (grey inset). (**b**) Percentage of variance explained by the preparatory (red) and movement

(green) subspaces. The left pair of bars corresponds to variance captured during the preparatory epoch. The right pair of bars corresponds to variance captured during the movement epoch. Stars denote significantly higher variance (p<0.001, one tailed test) with respect to 10-dimensional random subspaces (NS: not significant). Readers familiar with Kaufman *et al.*[1] may note an apparent difference between this figure and Figure 4a of Kaufman *et al*. despite using the same data. We point out that there is no inconsistency between these results. Kaufman *et al.* identified output-potent dimensions by regressing neural activity against muscle activity. In order to test their hypothesis, they performed their identification of subspaces blind to preparatory activity, and therefore likely did not identify their subspaces perfectly. Here, we perform our subspace identification informed by both preparatory and movement activity, and therefore could achieve better segregation of preparatory and movement subspaces.

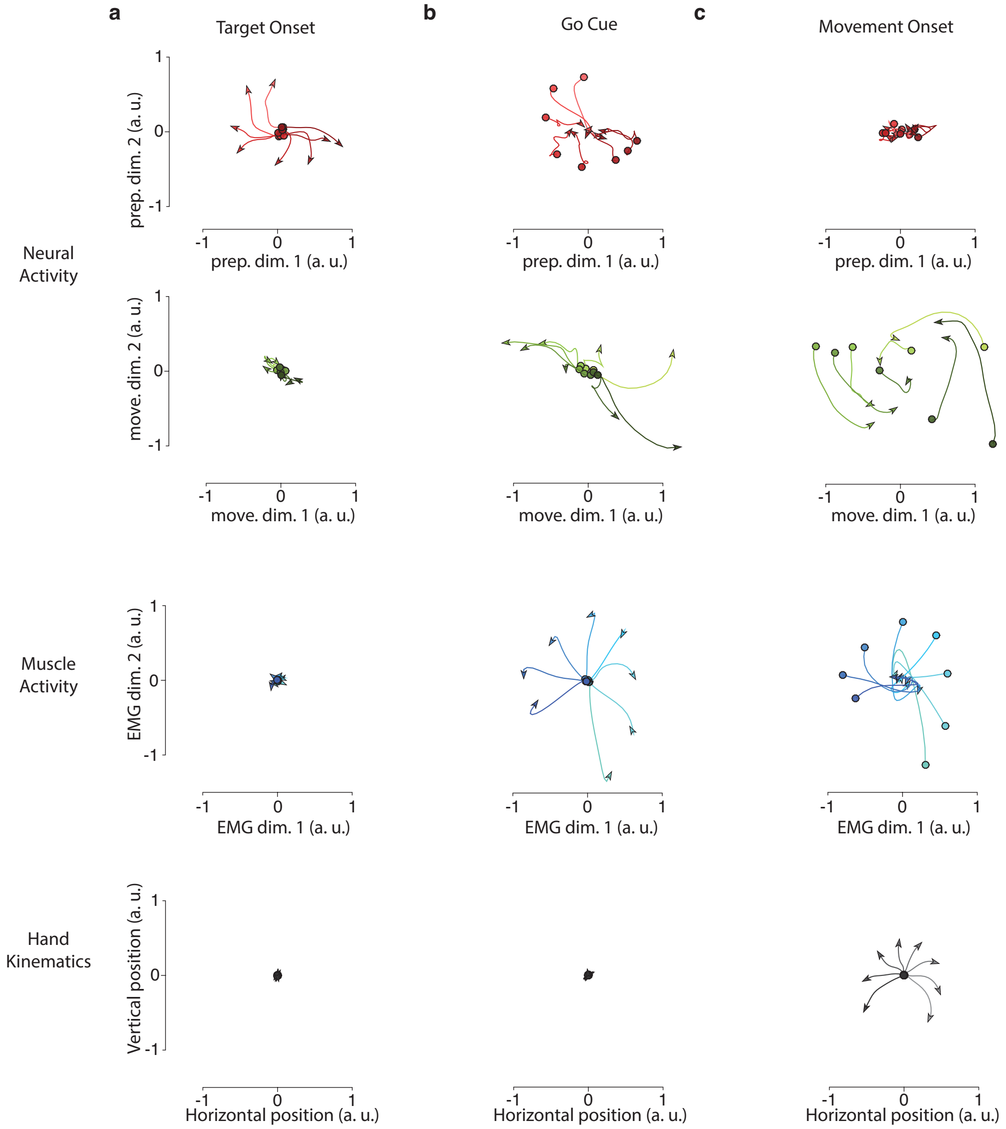

Supplementary Figure 8. Neural activity in four subspaces in response to three key task events (target onset, go cue, and movement onset).

This figure shows the same result as **Figure 6** from the main text but for monkey A. Each trace corresponds to a different reach direction. (**a**) Responses during a 150 ms window beginning at target onset. Data are shown for the neural population response (monkey A) projected onto two dimensions of the preparatory subspace (top), projected onto two dimensions of the movement subspace (second from top), for the top two principal components of muscle activity (second from bottom) and for hand position (bottom). (**b**) Same as in **a** but for response to go cue (during a 250ms window starting at the go cue and ending at approximately the onset of movement). (**c**) Same as in **a** but for a 200 ms window starting at movement onset.

Supplementary Note 1

Orthogonality implies, but is not implied by, a change in tuning

Across cortical systems, a mainstay of neural data analysis has been to consider selectivity or tuning[3,4] of single neurons. In the motor cortex, many studies have shown that the selectivity of individual neurons changes across different task contexts and across time[9,10]. In particular, selectivity often changes substantially between movement preparation and movement execution[11]. Thus, one naturally asks: is the orthogonality between the preparatory and movement neural responses a consequence of changing selectivity?

Here, we prove that change in selectivity does not imply that the population activity switches from one subspace to an orthogonal subspace. To do so, we construct a counterexample where data has zero average correlation between selectivity at one epoch relative to another epoch (different selectivity), yet the data in both epochs occupy a single neural subspace. The response subspace of dimensionality d may be defined by the top d principal components, which are the top left singular vectors of the data matrix. For the sake of this example, we define two toy responses at two epochs (P and M) that occupy the same neural subspace: let $P \in \mathbb{R}^{N \times C}$ be the preparatory response matrix, with $P = V\, S_{\mathrm{p}}\, U^T$, and $M \in \mathbb{R}^{N \times C}$ be the movement response matrix with, $M = V\, S_{\mathrm{m}}\, Z^T$ (C: number of conditions; N: number of neurons; U and $Z \in \mathbb{R}^{N \times d}$: right singular vectors for P and M, respectively; $V \in \mathbb{R}^{N \times d}$: the shared left singular vectors for P and M; S_{p} and S_{m} are the matrices of d nonzero singular values). Note by design, the left singular vectors of P and M are identical, reflecting the fact that they occupy the same single subspace.

The correlation of the selectivity of the i-th neuron responses between the two epochs (P_i: i-th row of P, and M_i: i-th row of M) is as follows:

$$\rho(P_i, M_i) = \frac{cov(P_i, M_i)}{\sigma_{P_i}\, \sigma_{M_i}} = \frac{\sum_{j=1}^{C}\left(P_{ij} - \bar{P}_i\right)\left(M_{ij} - \bar{M}_i\right)}{\sqrt{\sum_{j=1}^{C}\left(P_{ij} - \bar{P}_i\right)^2}\,\sqrt{\sum_{j=1}^{C}\left(M_{ij} - \bar{M}_i\right)^2}} = {P_i}^{\top} M_i$$

assuming for simplicity and without loss of generality that P_i and M_i are mean centered (*i.e.,* $\bar{P}_i = 0$ and $\bar{M}_i = 0$) and with unity variance (*i.e.,* $\sigma_{P_i} = 1$ and $\sigma_{M_i} = 1$). The average correlation of the selectivity between the two epochs over all neurons is then:

$$
\begin{aligned}
\bar{\rho} &= \frac{1}{N}\sum_{i=1}^{N} \rho(P_i, M_i) \\
&= \frac{1}{N}\sum_{i=1}^{N} P_i^{\top} M_i \\
&= \frac{1}{N} Tr(P^{\top} M) \\
&= \frac{1}{N} Tr\left(V S_{\mathrm{p}} U^{\top} Z S_{\mathrm{m}} V^{\top}\right) \\
&= \frac{1}{N} Tr\left(S_{\mathrm{p}} S_{\mathrm{m}} U^{\top} Z\right)
\end{aligned}
$$

The above expression depends on the dot product between the right singular vectors of P and M (U and Z). For example, if $Z = U$ the average correlation between the two responses is maximum ($\bar{\rho} = 1$). If $Z = -U$ the two responses will be negatively correlated ($\bar{\rho} = -1$). More importantly, if Z is in the null space of the columns of U the two responses will be uncorrelated ($\bar{\rho} = 0$). Thus, the responses P and M, which occupy the same neural subspace, may have perfectly matched selectivity or mismatched selectivity. Hence, selectivity mismatch does not imply orthogonal subspaces. To summarize, we can create datasets that occupy the same subspace but have arbitrary selectivity, which shows that differences in selectivity across computational epochs alone cannot explain the orthogonality between preparation and movement subspaces that we observe in the motor cortex.

Now we prove the converse that the orthogonality between the preparatory and movement subspaces does imply that neural selectivity between preparation and movement will be highly different. In other words, subspace orthogonality predicts that the preparation and movement selectivity will be uncorrelated on average. As before, we define two responses P and M. Unlike the above, P and M are now assumed to have orthogonal neural subspaces. In other words, $P = V S_{\mathrm{p}}\, U^{\top}$ and $M = Q\, S_{\mathrm{m}} Z^{\top}$, where $U,\ Z,\ S_{\mathrm{p}}$ and S_{m} are defined as before. V and Q are the left singular vectors, defining the neural subspaces. Before, Q was equal to V implying a single neural subspace, but now we

assume that Q is in the null-space of the columns of V. This assumption reflects the fact that neural subspaces of P and M are orthogonal. The expression for the mean selectivity correlation between P and M can be written as above:

$$\begin{aligned}\bar{\rho} &= \frac{1}{N}Tr(P^\top M)\\ &= \frac{1}{N}Tr\left(VS_\mathrm{p}U^\top ZS_\mathrm{m}Q^\top\right)\\ &= \frac{1}{N}Tr\left(Q^\top VS_\mathrm{p}U^\top ZS_\mathrm{m}\right)\\ &= 0\end{aligned}$$

Thus, if the neural responses occupy orthogonal subspaces, the selectivity of neurons across epochs is expected to be uncorrelated on average.

Supplementary Note 2

Coding model

The coding model stems from the classical view that the firing rates of neurons in the motor cortex are coding different kinematic factors. The neural responses during movement were a function of the hand position (p), velocity (v), acceleration (a), and jerk (j) with the following relative sensitivities (position: 12.5, velocity: 10, acceleration: 1, jerk: 0.05). Responses $r(t,c)$ at each time t and condition c were simulated by summing these kinematics factors at each time with weights (βs) drawn randomly from a uniform distribution in the range from -1 to 1 for each simulated neuron as follows:

$$\begin{aligned} r(t,c) = \; & \beta_{p_x} p_x(t,c) + \beta_{p_y} p_y(t,c) + \beta_p p(t,c) \\ & + \beta_{v_x} v_x(t,c) + \beta_{v_y} v_y(t,c) + \beta_v v(t,c) \\ & + \beta_{a_x} a_x(t,c) + \beta_{a_y} a_y(t,c) + \beta_a a(t,c) \\ & + \beta_{j_x} j_x(t,c) + \beta_{j_y} j_y(t,c) + \beta_j j(t,c) \end{aligned}$$

where p_x, p_y, and p are the position in the x direction, y directions and distance from reach starting point, respectively; v_x, v_y, and v are the velocity in the x direction, y directions and hand speed, respectively, and similar for $a_x,\ a_y,\ a,\ j_x,\ j_y,\ j$. Simulated preparatory activity was assumed to be proportional to horizontal reach end point, vertical reach end point, and maximum reach speed. This procedure will generate neurons with preferred directions in the kinematics space as specified in previous literature[3,4].

Generator model

The pattern generator model simulates an oscillatory dynamical system that generates muscle activity. This model has been previously proposed[2], and is presented in detail in Churchland *et al.*[2]. Two state space rotational planes with two different frequencies were simulated. These two rotational responses were summed with different weights to produce muscle activity (EMG). The oscillations were 90 degrees out of phase to mimic rotational trajectories in the state space, and were windowed by a gamma function. Different reaching conditions were simulated by running the system from a different initial condition, which corresponds to an oscillatory response with a different amplitude and

phase. However, the rotational frequencies and the relation between the leading and lagging responses remained consistent for all conditions. These initial states were extended back in time to simulate the preparatory neural activity. The initial conditions were optimized to fit the EMG activity at each condition via regression. The two oscillation frequencies, the parameters of the gamma windowing function, and the system evolution time were numerically optimized. The simulated neural responses were then a weighted sum of the underlying oscillations with weights drawn randomly.

Non-normal recurrent neural network (RNN1)

Simulated responses were generated from a recurrent neural network with non-normal connectivity matrix, which has been proposed previously in Hennequin *et al.*[5]. The weights matrix was initially sparse and random with half the columns positive only and half the columns negative only. Then, the inhibitory (negative) weights of the connectivity matrix were optimized according to the stability optimization procedure discussed in Hennequin *et al.* This optimization procedure maintains the non-normal structure of the network[6] while stabilizing the network's chaotic activity. Refer to Hennequin *et al.* for full details of the model and the stability optimization procedure. The simulated preparatory activity was generated from holding the network activity at an initial fixed point, and the simulated movement activity was generated by freely running the network from this initial fixed point. The initial fixed point was different for each reaching condition. These initial conditions were chosen to generate network responses with energy that matched the real evoked neural energy (the square norm of the vector of firing rates at each time) as follows: first, we identified a ranked dictionary of N preferred states (N: number of units in the network) that evoke the most to the least energy of the network. In other words, the top preferred state ($\mathbf{x}_1 \in \mathbb{R}^N$) evokes the highest neural energy in the network; the second preferred state ($\mathbf{x}_2 \in \mathbb{R}^N$) evokes the second highest neural energy, and so on. For a condition c, we assumed that the network initial fixed point at that condition ($\mathbf{r}(0, c) \in \mathbb{R}^N$) is a weighted sum of all the preferred states. To obtain the corresponding weights, for each condition, we measured the correlation coefficient between the real neural energy and the model energy evoked from each of the N preferred states. We then picked the top 5 preferred states that evoked energy similar to the real data and weighted them as follows:

$$\mathbf{r}(0, c) = [\mathbf{x}_1\ \mathbf{x}_2\ \dots\ \mathbf{x}_N] \frac{\mathbf{w}_c}{\|\mathbf{w}_c\|_2}$$

where $\mathbf{w}_c \in \mathbb{R}^N$ is the vector that contains the top 5 correlation coefficients of the network energy with the real data energy at condition c. These initial conditions generated network responses with similar energy to the real data. In addition, the network responses were rich enough to reproduce real muscle activity (EMG) with a simple linear read out.

Regularized recurrent neural network model (RNN2)

Simulated responses were generated from another randomly connected recurrent neural network, described in full detail in Sussillo *et al.*[7]. The network responses were trained to generate EMG activity while regularizing the network responses to be as simple and smooth dynamics as possible. The network activity was held at a fixed point during the preparatory epoch. Then, a go signal turned on strong network dynamics simulating the movement activity. Here we used the simulated data from Sussillo *et al.*

Supplementary Note 3

Sampling random dimensions in the space of neural data

Here we aim to draw random subspaces biased to the data covariance structure. The assumption here is that there is a fixed correlation between neurons that governs the neural responses at all times[8]. This fixed correlation implies a fixed space where computations can be performed. Our goal is to test if the orthogonality between specific neural subspaces, such as preparatory and movement, is beyond what is expected from randomly sampled subspaces within this space. Sampling directions uniformly in neural space will depend mainly on the number of neurons recorded, not the correlation structure of the data. To overcome this difficulty, we developed a Monte Carlo analysis that generated random subspaces aligned to the space occupied by neural data (preserving this correlation structure). We first calculated the covariance matrix (C) from real neural responses across all times of the task. We then sampled random subspaces ($\mathbf{v}_{\text{align}}$) aligned to the structure of neural data space according to this static covariance, as follows:

$$\mathbf{v}_{\text{align}} = \boldsymbol{orth}\left(\frac{U\sqrt{S}\mathbf{v}}{\left\|U\sqrt{S}\mathbf{v}\right\|_2}\right)$$

where U and S are the eigenvectors and eigenvalues matrices of C, respectively. $\mathbf{v} \in \mathbb{R}^{N\times d}$ is a matrix with each element drawn independently from a normal distribution with mean zero and variance one. $\boldsymbol{orth}(Z)$ returns the orthonormal basis of the matrix Z defined by its left singular vectors. This procedure samples subspaces biased towards the space of neural activity, such that the sampled random subspaces will have the specified neuronal covariance structure C (**Supplementary Fig. 3**). To calculate the distribution of alignment indices of any two 10-dimensional random subspaces $(d = 10)$, we sampled two sets of random dimensions ($\mathbf{v}_{\text{align}}^{(i)}$ and $\mathbf{v}_{\text{align}}^{(j)}$). The alignment index is then $A_{ij} = \frac{\boldsymbol{Tr}\left(\mathbf{v}_{\text{align}}^{(j)\top}\mathbf{v}_{\text{align}}^{(i)}\mathbf{v}_{\text{align}}^{(i)\top}\mathbf{v}_{\text{align}}^{(j)}\right)}{d}$. The distribution of random alignment indices (Main text **Figure 4c**) is obtained from repeating this sampling procedure 10000 times.

Supplementary References

1 Kaufman, M. T., Churchland, M. M., Ryu, S. I. & Shenoy, K. V. Cortical activity in the null space: permitting preparation without movement. *Nature Neuroscience* **17**, 440-448, doi:DOI 10.1038/nn.3643 (2014).

2 Churchland, M. M. *et al.* Neural population dynamics during reaching. *Nature* **487**, 51-+, doi:DOI 10.1038/nature11129 (2012).

3 Moran, D. W. & Schwartz, A. B. Motor cortical representation of speed and direction during reaching. *Journal of Neurophysiology* **82**, 2676-2692 (1999).

4 Georgopoulos, A. P., Kalaska, J. F., Caminiti, R. & Massey, J. T. On the Relations between the Direction of Two-Dimensional Arm Movements and Cell Discharge in Primate Motor Cortex. *Journal of Neuroscience* **2**, 1527-1537 (1982).

5 Hennequin, G., Vogels, T. P. & Gerstner, W. Optimal control of transient dynamics in balanced networks supports generation of complex movements. *Neuron* **82**, 1394-1406, doi:10.1016/j.neuron.2014.04.045 (2014).

6 Murphy, B. K. & Miller, K. D. Balanced Amplification: A New Mechanism of Selective Amplification of Neural Activity Patterns. *Neuron* **61**, 635-648, doi:10.1016/j.neuron.2009.02.005 (2009).

7 Sussillo, D., Churchland, M. M., Kaufman, M. T. & Shenoy, K. V. A neural network that finds a naturalistic solution for the production of muscle activity. *Nat Neurosci* **18**, 1025-1033, doi:10.1038/nn.4042 (2015).

8 Sadtler, P. T. *et al.* Neural constraints on learning. *Nature* **512**, 423-U428, doi:10.1038/nature13665 (2014).

9 Kaufman, M. T. *et al.* Roles of monkey premotor neuron classes in movement preparation and execution. *J Neurophysiol* **104**, 799-810, doi:10.1152/jn.00231.2009 (2010).

10 Crammond, D. J. & Kalaska, J. F. Prior information in motor and premotor cortex: activity during the delay period and effect on pre-movement activity. *J Neurophysiol* **84**, 986-1005 (2000).

11 Churchland, M. M. & Shenoy, K. V. Temporal complexity and heterogeneity of single-neuron activity in premotor and motor cortex. *Journal of Neurophysiology* **97**, 4235-4257, doi:DOI 10.1152/jn.00095.2007 (2007).
